# Transinfection of *Wolbachia w*AlbB into *Culex quinquefasciatus* mosquitoes does not alter vector competence for Hawaiian avian malaria (*Plasmodium relictum* GRW4)

**DOI:** 10.1371/journal.ppat.1012052

**Published:** 2024-08-05

**Authors:** A. Marm Kilpatrick, Christa M. Seidl, Isaiah J. Ipsaro, Chris E. Garrison, Giulia Fabbri, Paul I. Howell, Austin G. McGowan, Bradley J. White, Sara N. Mitchell

**Affiliations:** 1 Department of Ecology and Evolutionary Biology, University of California, Santa Cruz, California, United States of America; 2 Verily Life Sciences, South San Francisco, California, United States of America; Institut Pasteur, FRANCE

## Abstract

Avian malaria is expanding upslope with warmer temperatures and driving multiple species of Hawaiian birds towards extinction. Methods to reduce malaria transmission are urgently needed to prevent further declines. Releasing *Wolbachia*-infected incompatible male mosquitoes could suppress mosquito populations and releasing *Wolbachia*-infected female mosquitoes (or both sexes) could reduce pathogen transmission if the *Wolbachia* strain reduced vector competence. We cleared *Culex quinquefasciatus* of their natural *Wolbachia pipientis w*Pip infection and transinfected them with *Wolbachia w*AlbB isolated from *Aedes albopictus*. We show that *w*AlbB infection was transmitted transovarially, and demonstrate cytoplasmic incompatibility with wild-type mosquitoes infected with *w*Pip from Oahu and Maui, Hawaii. We measured vector competence for avian malaria, *Plasmodium relictum*, lineage GRW4, of seven mosquito lines (two with *w*AlbB; three with natural *w*Pip infection, and two cleared of *Wolbachia* infection) by allowing them to feed on canaries infected with recently collected field isolates of Hawaiian *P*. *relictum*. We tested 73 groups (N_total_ = 1176) of mosquitoes for *P*. *relictum* infection in abdomens and thoraxes 6–14 days after feeding on a range of parasitemias from 0.028% to 2.49%, as well as a smaller subset of salivary glands. We found no measurable effect of *Wolbachia* on any endpoint, but strong effects of parasitemia, days post feeding, and mosquito strain on both abdomen and thorax infection prevalence. These results suggest that releasing male *w*AlbB-infected *C*. *quinquefasciatus* mosquitoes could suppress *w*Pip-infected mosquito populations, but would have little positive or negative impact on mosquito vector competence for *P*. *relictum* if *w*AlbB became established in local mosquito populations. More broadly, the lack of *Wolbachia* effects on vector competence we observed highlights the variable impacts of both native and transinfected *Wolbachia* infections in mosquitoes.

## Author summary

Avian malaria is expanding upslope in Hawaii with warmer temperatures and driving multiple species of birds towards extinction. Methods to reduce malaria transmission are urgently needed to prevent further declines. Releasing male mosquitoes infected with a type of bacteria (*Wolbachia*) could suppress mosquito populations by reducing offspring viability and releasing *Wolbachia*-infected female mosquitoes (or both sexes) could reduce pathogen transmission if the *Wolbachia* strain reduced vector competence. We cleared *Culex quinquefasciatus* of their natural *Wolbachia pipientis w*Pip infection and transinfected them with *Wolbachia w*AlbB isolated from *Aedes albopictus*. We show that *w*AlbB infection was transmitted to offspring, and mating *w*AlbB males with wild-type mosquitoes produced mostly inviable offspring. We measured susceptibility to avian malaria infection of mosquitoes with native or transinfected *Wolbachia* (*w*Pip or *w*AlbB). We found no measurable effect of *Wolbachia* on susceptibility. These results suggest that releasing male *w*AlbB-infected *C*. *quinquefasciatus* mosquitoes could suppress Hawaiian mosquito populations, but would have little positive or negative impact on mosquito vector competence for avian malaria. More broadly, the lack of *Wolbachia* effects on susceptibility to avian malaria we observed highlights the variable impacts of both native and transinfected *Wolbachia* infections in mosquitoes.

## Introduction

Hawaiian birds are experiencing an extinction crisis. Of the 47 species of Hawaiian honeycreepers known to science, forty-one are extinct or federally endangered [1]. One of the key threats to the remaining species is avian malaria, *Plasmodium relictum* (lineage GRW4), transmitted by *Culex quinquefasciatus* mosquitoes [1–4]. Rising global temperatures due to climate change have increased the distribution of mosquito populations and the transmission of malaria at higher elevations on multiple islands [5], as predicted two decades ago [6]. This has led to further population declines in many species [7], with two additional species now nearly extinct in the wild [1]. New tools are urgently needed to reduce transmission of avian malaria to prevent species extinctions in the wild, and allow populations to recover.

One recent breakthrough for reducing transmission of mosquito-borne diseases is the use of *Wolbachia* bacteria, either to suppress mosquito populations or to reduce mosquito vector competence [8,9]. *Wolbachia* bacteria are intracellular organisms that naturally infect many mosquitoes and insect species [10], and they can be transinfected into new species or populations [11]. Different strains of *Wolbachia* can have different effects on mosquitoes, including reduced adult lifespan or fecundity, and reduced vector competence for some pathogens [12]. In addition, *Wolbachia* can result in inviable embryos through cytoplasmic incompatibility (CI) if male and female mosquitoes have incompatible *Wolbachia* strains or males have *Wolbachia* but females do not [12]. Cytoplasmic incompatibility can be used to reduce mosquito populations by releasing incompatible males, or to drive *Wolbachia* into a population by providing an advantage to females that carry it [12,13]. Control strategies involving the release of *Wolbachia*-infected incompatible males could further reduce pathogen transmission if the *Wolbachia* strain in accidentally released female mosquitoes [14,15] also reduces vector competence [16]. Similarly, if a *Wolbachia* strain reduces vector competence, then population replacement via large scale releases of female (and male) mosquitoes infected with this *Wolbachia* strain could reduce vector competence and transmission, as has been recently demonstrated for dengue virus and *Aedes aegypti* mosquitoes [17,18]. The EPA recently approved an emergency exemption to release large numbers of *w*AlbB transinfected male mosquitoes to reduce populations of *C*. *quinquefasciatus* in Hawaii [19], eliminating one regulatory barrier for using this strategy to decrease transmission of avian malaria in Hawaii.

Our goals were threefold: first, to transinfect *Wolbachia w*AlbB into a *C*. *quinquefasciatus* line to use for incompatible male releases for population suppression in Hawaii; second, to confirm complete transmission of *Wolbachia* infection and CI of transinfected males with wild-type females from Hawaii infected with *Wolbachia w*Pip; and third, to determine if the transinfected *Wolbachia w*AlbB strain alters the vector competence of transinfected female *C*. *quinquefasciatus* mosquitoes for the avian malaria lineage found in Hawaii, *P*. *relictum* GRW4. We did not have an *a priori* hypothesis about whether the *w*AlbB strain of *Wolbachia* would increase or reduce vector competence of *C*. *quinquefasciatus* for *P*. *relictum* because effects of transinfected *Wolbachia* in other mosquito species have been highly variable [9,20] and no studies of the effects of transinfected *Wolbachia* on malaria competence have been done in *C*. *quinquefasciatus*. Previous studies of *C*. *quinquefasciatus* naturally infected with *Wolbachia w*Pip found higher susceptibility for *P*. *relictum* lineage SGS1 than mosquitoes cleared of *w*Pip with antibiotics [21]. However, there was no effect of *Wolbachia w*Pip on infection prevalence in the field [22]. Finally, a previous study that created a *w*AlbB-transinfected *C*. *quinquefasciatus* line didn’t quantify the effects on vector competence for any pathogen [23], and this line has been lost in an insectary malfunction (Steven Sinkins, personal communication).

## Methods

### *Culex quinquefasciatus* mosquito strains

We studied eight *C*. *quinquefasciatus* mosquito lines (Table A in S1 Appendix) which were combinations of four mosquito strains (Palmyra Atoll (abbreviated Palmyra or Palm in figures), Oahu, Maui, and Field (Captain Cook, Hawaii)) with one of two *Wolbachia* strains (native *w*Pip or transinfected *w*AlbB) or cleared of *Wolbachia* infection via antibiotic treatment (“None”). We refer to lines using the strain and *Wolbachia* type (e.g. Palmyra-*w*Pip, or Oahu-None). We used subsets of these eight lines for three types of experiments: inheritance of transinfected *w*AlbB *Wolbachia*, cytoplasmic incompatibility, and vector competence for avian malaria, *P*. *relictum* GRW4 (Table A in S1 Appendix).

The Palmyra-*w*Pip strain was started from egg rafts collected from Palmyra Atoll (5.889481°N, 162.078570°W, 5 m above sea level) in 2018 and reared for 40 generations in the laboratory. We created a second line (Palmyra-None) by clearing the Palmyra-*w*Pip line of *Wolbachia* for 5 generations using antibiotics (see below). We created a third line by transinfecting the Palmyra-None line with *wAlbB* (Palmyra-*w*AlbB) (see below).

The Oahu-*w*Pip strain was started from eggs collected from Kawainui Marsh (21.393988°N, 157.752154°W, 1 m above sea level) in 2020 and reared for 10 generations. We created the Oahu-None line by clearing the Oahu-*w*Pip line with antibiotics 5 generations. We created the Oahu-*w*AlbB line by backcrossing the Oahu-None line with the Palmyra-*w*AlbB line for seven generations.

The Maui-*w*Pip strain was started using egg rafts collected from Makawao, HI (20.851838°, -156.313887°, 499 m above sea level) in 2020. Finally, the Field-*w*Pip line was started using egg rafts collected from Captain Cook, Hawaii (19.461187°N, 155.896432°W, 204m above sea level) in March 2023; for this line, larvae were reared and G0 individuals were used for measuring vector competence (Table A in S1 Appendix).

All 7 colonized lines (all but Field-*w*Pip) were reared using standard protocols [14], with 8–10 egg rafts seeded into each 1 Liter rearing pan; adults were fed on avian blood (Lampire Biological Laboratories); and females were allowed to lay eggs directly on reverse osmosis (RO) water. All mosquito stages were maintained at 26–28 °C in 70–80% humidity. Adult mosquitoes 5–10 days old from the 7 colonized lines were transported by car from Verily’s rearing facility to the University of California, Santa Cruz (~110 kilometers) for the vector competence studies and fed on birds in the subsequent 36 hours. We reared Field-wPip larvae in plastic pans (44 cm × 25 cm × 10 cm) with 200–350 larvae/pan, filled with 1 Liter of deionized water and fed daily 0.2–0.4 g of ground fish food (Koi’s Choice Premium Fish Food). We transferred pupae to 30 cm^3^ mosquito cages (BugDorm). Emerging adults were fed *ad libitum* on 10% sucrose solution-soaked cottons. We used 7 to 14-day-old Field-*w*Pip adults and deprived them of sucrose cottons for 24–48 hrs prior to blood feeding in vector competence experiments.

### Clearing *Wolbachia w*Pip, transinfecting *Wolbachia w*AlbB

We created two mosquito lines without *Wolbachia*, Palmyra-None and Oahu-None, by clearing native *Wolbachia w*Pip with antibiotics and then used these two lines to create two additional mosquito lines transinfected with *Wolbachia w*AlbB. We first cleared native *Wolbachia w*Pip infection from the Palmyra-*w*Pip line by exposing adult mosquitoes to a solution of 25mg/100ml tetracycline (Sigma T7660), pH buffered with TrisBase to 7.4, and provided in 10% sugar feeders for five generations [24]. We then isolated *Wolbachia w*AlbB from *Aedes albopictus* originating from Kuala Lumpur, Malaysia (KLP strain). We dissected 30–60 ovaries of female KLP mosquitoes and harvested *w*AlbB *Wolbachia* into phosphate buffered saline (PBS) for each transfection experiment. Ovary tissue was thoroughly homogenized using a Tapered Tissue Grinder (DWK Life Sciences Wheaton^™^) before centrifugation to remove cellular debris. We filtered and centrifuged the supernatant (Pall Acrodisc) to select for *Wolbachia* cells. We resuspended the pellet in PBS to a concentration of 1–6 ovaries/μL for microinjection into *Culex* embryos.

We then transinfected mosquitoes from the Palmyra-None line with *w*AlbB. We allowed Palmyra-None females to lay eggs on RO water and aligned eggs individually against nitrocellulose membrane (MF-Milipore #HAWP04700) underneath moistened filter paper on a glass slide. We then injected *w*AlbB *Wolbachia* extract into each egg at the posterior end using a beveled quartz glass pipette (Sutter Instruments #QF100-70-10) and the XenoWorks Digital Microinjector and Micromanipulator system (Sutter Instruments).

Post injection, we stored *w*AlbB eggs in a plastic container with a constant RO water supply and incubated overnight at 28°C before hatching the next day. We hatched injected eggs in a petri dish with RO water, yeast slurry and Koi fish pellets. We maintained flooded eggs under insectary conditions (28°C, 80% humidity, 12:12 hr light cycle) for ~3 days before larvae were counted. We reared larvae to adults on a diet of Koi fish pellets.

We harvested adult males from G0 (transinfected eggs) and screened them for the presence of *Wolbachia* using ddPCR [14] to detect transinfection. We mated females from injection sets with positive G0 males with Palmyra-None males and provided them with a warmed avian blood meal in plastic 100 x 15 mm petri-dishes (VWR, #25384–324) with Parafilm M membrane (Bevis, #HS234526A). Isofemale lines were then established from single blood fed females placed into iso-tubes (Drosophila vials [Genesee Scientific 32–116]) with RO water (~10 mL). Once eggs were laid, females were harvested and individually tested for the presence of *Wolbachia* via ddPCR. If an egg-laying female was positive for *Wolbachia*, its G1 larvae were reared and the process of male and female screening repeated until a stable line with 100% infection was achieved.

### Transmission of transinfected *w*AlbB

We examined transmission of *w*AlbB in Palmyra-*w*AlbB (“DQB”, *D*ebug *q*uinquefasciatus *w*Alb*B*.”) mosquitoes and confirmed the absence of both *w*AlbA and *w*Pip in the Palmyra-*w*AlbB line. We stabilized the Palmyra-*w*AlbB line through sib-mating and standard larva-to-adult rearing for six generations before *Wolbachia* transmission was assessed. We then quantified transmission of *w*AlbB *Wolbachia* in at least 95 adult males and 95 females in each of five generations (G7, 8, 10, 11, 12; G9 had insufficient samples). We extracted mosquito DNA and quantified *w*AlbB presence and *w*AlbA and *w*Pip absence using ddPCR on the *WSP* gene with established *w*AlbB primer probes [14], *w*AlbA primer probes (F 5’CCCCAGCAGATACTATTGCG 3’, R 5’ CGGTTTTCAAAGGAGTGCTG 3’, probe 5’ 6-FAM TTGGTGTTGGTGTTGGTGCAGCGT ZEN/3’ IBFQ) and *w*Pip primer probes (F 5’ GCTGGTGCTCGTTATTTTGG 3’, R 5’ ACAGCGCTGTAAAGGACATT 3’, probe 5’ 6-FAM AAGAAGCAGTATCAGCTACTAAAGAGA ZEN/3’ IBFQ). We made the following modifications: we used the following for the ddPCR primer probe set for the *C*. *quinquefasciatus* housekeeping gene *RPL5* (ensembl ID LOC6043019): F 5’ GGCAACACTGACATCTCTGT 3’, R 5’ AAAATCGAAGATGGCGTTGC 3’, probe 5’ HEX CTCGCTCGCTCTCGGCTCTCCT ZEN/3’ IBFQ. A few samples (15/991) were omitted from analysis because the mosquito reference gene *RPL5* was less than 100 droplets, which indicated poor DNA quality/yield.

### Bidirectional cytoplasmic incompatibility and fecundity

We examined bidirectional CI between Palmyra-*w*AlbB and both Oahu-*w*Pip and Maui-*w*Pip. Bidirectional CI occurs when crosses in both directions (male *w*Pip and female *w*AlbB and vice versa) both result in inviable offspring. We assessed CI in the Palmyra-*w*AlbB transinfected line by mating Palmyra-*w*AlbB males to two lines with native *w*Pip infections, Oahu-*w*Pip and Maui-*w*Pip (Table A in S1 Appendix). We measured bidirectional CI by mating Oahu-*w*Pip and Maui-*w*Pip males with Palmyra-*w*AlbB females. We assessed sex of pupae microscopically before transfer to eclosion cages (BugDorm-4S3030, W30 x D30 x H30cm) provisioned with of 10% sucrose *ad libitum*. Four days after eclosion, when mosquitoes were sexually mature, we mated 150 males from one of two *Wolbachia* strains (*w*AlbB or *w*Pip) with 100 females of the other *Wolbachia* strain (*w*Pip or *w*AlbB, respectively) in medium cages (BugDorm-4S3030, W30 x D30 x H30cm) for three nights. After mating, we provided females an avian blood meal (Lampire Biological Laboratories) in a plastic 100 x 15mm petri-dish (VWR, #25384–324) with Parafilm M membrane (Bevis, #HS234526A). Following blood feeding, we allowed females to oviposit individually in iso-tubes (Genesee Scientific 32–116) with RO water (~10 mL) or *en masse* in oviposition bowls (Eco-Products, EP-BSC8-GS) with ~ 100 mL of RO water. We collected rafts and counted eggs under the microscope before hatching, and returned them to RO water to hatch for seven days before any larvae were counted. For each of the four mating crosses (Palmyra-*w*AlbB males with female Oahu-*w*Pip and Maui-*w*Pip; and Palmyra-*w*AlbB females with male Oahu-*w*Pip and Maui-*w*Pip) three biological replicates from different mosquito generations were performed.

We also measured fecundity (# of eggs laid per female) for the transinfected Palmyra-*w*AlbB and Oahu-*w*AlbB strains, two wild-type strains that we used for CI testing (Maui-*w*Pip and Oahu-*w*Pip), and crosses between mosquitoes infected with *w*Pip and *w*AlbB described above. We placed a subset of 29–96 individual females from each strain into iso-tubes after blood feeding for egg laying as described above. We added 1 drop of yeast solution to each egg raft in the iso-tubes.

### *Plasmodium relictum* isolates

We collected two isolates of *Plasmodium relictum* (lineage GRW4) from wild birds at two sites on Hawai’i Island, one from an ‘Apapane (*Himatione sanguinea*) from Pu’u Wa’awa’a Forest Reserve (19.738154°N, 155.875234°W, 1,230 m above sea level) and another from a Warbling White-eye (*Zosterops japonicus*) from the same Captain Cook, HI site described above where Field mosquitoes were collected. We took 50–100 μl of blood from the brachial vein of birds and placed it into a 1 mL syringe with an appropriate volume of citrate–phosphate–dextrose solution with adenine (CDPA; Sigma-Aldrich, St. Louis, MO, USA) to create a 1:9 CDPA to blood ratio [25] and stored it up to 48 hrs at 4 °C. We used 1–2 μl of blood from the same bird to create a thin blood smear that was air-dried for 30 minutes, fixed in 100% methanol, and later stained for one hour with Giemsa [26]. We screened each stained smear by examining 50 microscope fields at 1000x magnification using oil immersion to determine the presence and parasitemia of a sample. If we observed one or more cells infected with malaria, we inoculated the blood-CDPA mixture intramuscularly into the pectoral muscle of a domestic canary (*Serinus canaria*). We passaged isolates one to seven times in canaries via intramuscular inoculations of 50–100 μl of infected blood between birds before exposure to mosquitoes in feeding trials, or before cryopreservation in glycerolyte for future use [27]. Both isolates used in mosquito feedings had at least 1 passage between the wild source bird and the bird used for mosquito feedings. All work with wild and laboratory birds was performed under animal care and use protocol Kilpm2003 approved by the Institutional Animal Care and Use Committee at the University of California in Santa Cruz, USA.

### Experimental *Plasmodium relictum* infections, mosquito feeding and survival

We inoculated canaries intramuscularly with 50–200 μL of whole blood (parasitemia: 0.1–2.85%) containing an avian malaria isolate that had been passaged 1–7 times and was either a thawed deglycerolized sample, or was fresh blood from another infected canary. Starting on day 5 post-infection afterward we took 5–10 μL of blood by brachial venipuncture and screened thin blood smears by microscopy and by qPCR [28,29] to detect infection and estimate parasitemia. Once infection was detected, we allowed approximately 50 mosquitoes from each of three mosquito lines to feed on each infected bird simultaneously. We differentiated the three mosquito lines during feedings by spraying each line with a green or red fluorescent marker or leaving it unmarked (Fig A and Methods in S1 Appendix); the line receiving each spray color (or none) was randomly selected. We fed most mosquitoes by restraining an infected canary on top of a container containing the mosquitoes that had holes in the lid that allowed the bird’s legs and feet to be inside the container (Fig B in S1 Appendix). Field mosquitoes were more hesitant to feed and were placed in a Bugdorm with an unrestrained bird in a PVC cylinder with a perch and allowed to feed for ~ 8 hrs overnight (Fig B in S1 Appendix).

We collected engorged mosquitoes with an aspirator and transferred them into a cage in an incubator set to 26 °C. We provided mosquitoes with cottons soaked in a 10% sucrose solution and held them until dissection. We recorded deaths, daily, of all adult females and assigned them to a mosquito line based on the spray color, whenever possible. We collected unfed females, knocked them down on ice, and counted the number of each spray color under a UV light to quantify feeding success for each group.

We killed engorged mosquitoes 6–14 days after feeding and dissected them by cleanly separating the thorax from the abdomen at the scutellum with sterile dissection needles. We placed abdomens in 96-well DNA extraction plates containing Chemagic DNA lysis buffer (PerkinElmer, Waltham, MA, USA) and placed the head, thorax, and legs of each individual in a second plate to test for a disseminated sporozoite infection which we call “thorax infection”. When DNA extraction plates were not available, abdomens and thoraxes (including the head and legs) were placed in separate 1 mL vials containing 0.5 mL of 70% ethanol. All samples were stored at -20 °C until DNA extraction and processing by qPCR (Methods: *Plasmodium relictum* qPCR and ddPCR in S1 Appendix). We also examined *P*. *relictum* infection in the salivary glands for a subset of 106 Oahu mosquitoes of all three Wolbachia types (*w*AlbB, *w*Pip, and None) (Methods: Salivary gland infection in S1 Appendix). We were not able to measure *Plasmodium* infection in salivary glands for all mosquitoes which would have enabled us to determine the fraction of mosquitoes with infective sporozoite thorax infections that were infectious and would likely transmit malaria. We also note that *P*. *relictum*-positive abdomens may have contained oocysts or other malaria stages since we did not dissect midguts.

### Statistical analyses

We analyzed egg viability (and cytoplasmic incompatibility) among crosses, *Wolbachia* types, and mosquito strains using a generalized linear model with a binomial distribution and a logit link. We analyzed fecundity with a generalized linear model with a negative binomial distribution and a log link. We analyzed adult female survival in the 11 days post-blood feeding with a Cox’s proportional hazard model, which censored the mosquitoes that were killed for testing. We analyzed the fraction of mosquitoes testing positive for *P*. *relictum* by qPCR with generalized linear models with a binomial distribution and a logit link. We analyzed abdomen and thorax infections separately. We included log(Parasitemia), days post feeding, mosquito strain (Oahu, Palmyra, or Field), and *Wolbachia* type (*w*AlbB, *w*Pip, or None) as predictors. We excluded small batches of mosquitoes with N<4 (a batch is a unique mosquito strain + *Wolbachia* strain + day post feeding + parasitemia), but results were qualitatively identical if we included all mosquitoes. We examined two and three-way interactions among the predictors log_10_(Parasitemia), days post feeding, and mosquito strain and used AIC to determine which interactions were best supported by the data. We did not include spray color or *P*. *relictum* isolate in the final model because neither had support when added to the best fitting model (Table I in S1 Appendix; Spray color: χ^2^ = 1.47, df = 2, P = 0.48; Pu’u Wa’awa’a Isolate coef.: -0.225, SE = 0.18, Z = -1.23, P = 0.22).

To analyze salivary gland infection, we log transformed the ratio of *P*. *relictum* DNA to mosquito DNA in salivary gland samples and analyzed it with a linear model with *Wolbachia* type as a predictor. There was heteroscedasticy in the residuals so we performed a robust comparison using the *coeftest* function in the *lmtest* package with a variance-covariance matrix estimated using the *sandwich* package. All analyses were performed in R, v.4.3.3. Data and code to replicate the analyses and figures is available from [30].

## Dryad DOI

10.5061/dryad.k6djh9wf4 [30].

## Results

### Transinfected line *Wolbachia* transmission and Cytoplasmic Incompatibility (CI)

We cleared *w*Pip *Wolbachia* infection from the Palmyra-*w*Pip strain of *C*. *quinquefasciatus* with antibiotics. Zero of 60 mosquitoes (48 females 12 males) from the antibiotic treated Palmyra line and zero of 90 mosquitoes from the antibiotic treated Oahu line (45 females 45 males) tested positive for *w*Pip by ddPCR while 100% of these 150 mosquitoes tested positive for a control gene (RPL5). We then successfully transinfected a single female cleared of *w*Pip with the *w*AlbB strain of *Wolbachia*. We examined transmission in 991 adults from generations 7 thru 13 of the mosquito line from this female (N_ave_ = 188.8 (range 187–192)/generation, excluding generation 9 which had 32 mosquitoes). Every sample from every generation tested positive for *w*AlbB by ddPCR, indicating complete transmission of *Wolbachia* across generations (mean ratio of wAlbB gene copies to *C*. *quinquefasciatus* gene copies: 2.95; SE = 0.077; Fig C in S1 Appendix). None of the mosquitoes tested positive for *w*AlbA (Fig C in S1 Appendix).

**Fig 1:**
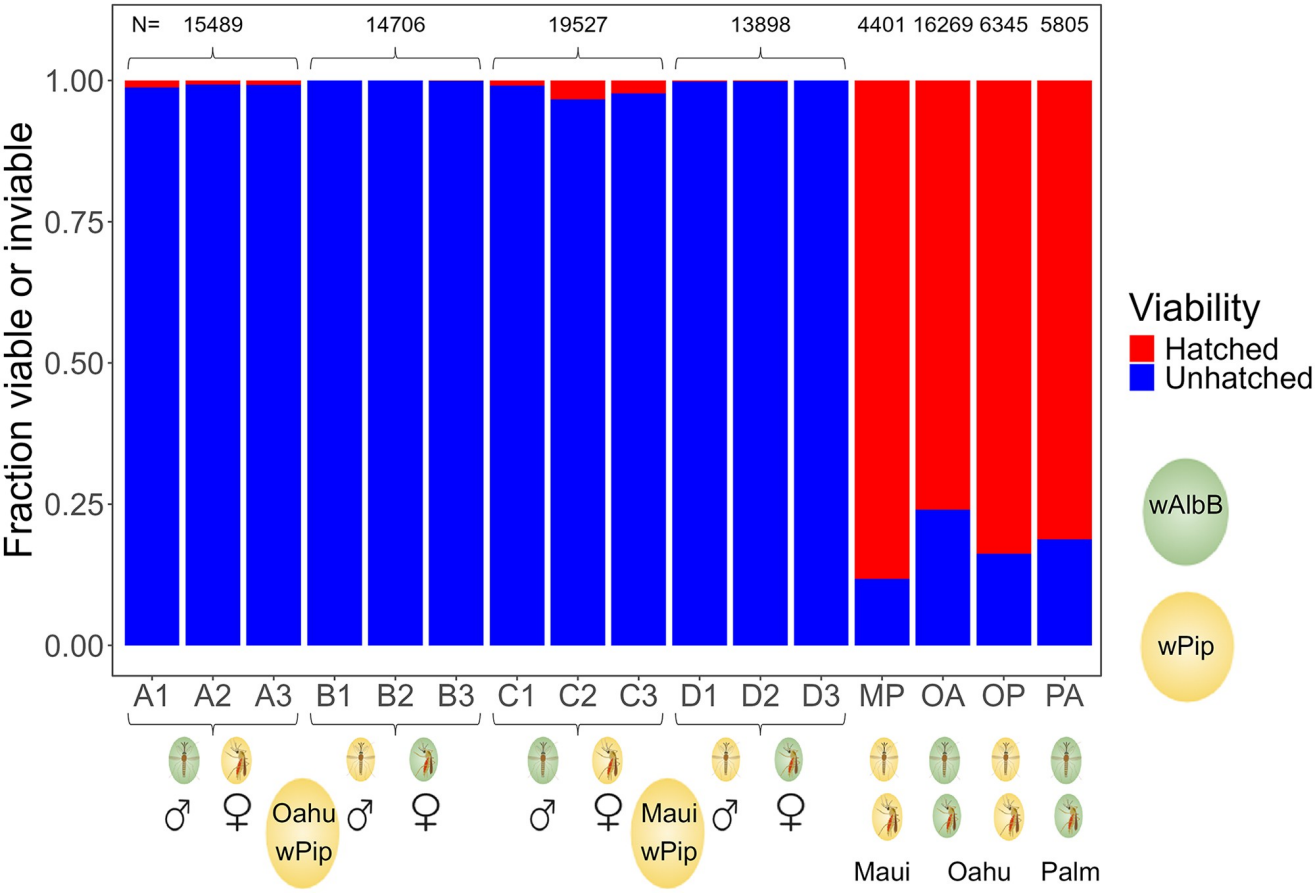
Bi-directional cytoplasmic incompatibility results between mosquitoes infected with *Wolbachia wAlbB* (green ovals) and two mosquito strains (Oahu and Maui) infected with native *Wolbachia wPip* (yellow ovals) and viability for compatible crosses. Three replicates (1–3) of eggs from an average of 36.1 (range 17–45) females/replicate are shown for each of four mixed *w*AlbB-*w*Pip crosses (A, B, C, D) that include males and females of each mosquito strain (Tables C and D in S1 Appendix): A1-A3: Palmyra-*w*AlbB males X Oahu-*w*Pip females; B1-B3: Oahu-*w*Pip males X Palmyra-*w*AlbB females; C1-C3: Palmyra-*w*AlbB males X Maui-*w*Pip females; D1-D3: Maui-*w*Pip males X Palmyra-*w*AlbB females. Each of the 12 replicates A1-D3 had N_mean_ = 4796 (range 786–7131) eggs. The four remaining crosses were between male and female mosquitoes with the same *Wolbachia* type (MP: Maui-*w*Pip, OA: Oahu-*w*AlbB, OP: Oahu-*w*Pip, PA: Palmyra-*w*AlbB (Table E in S1 Appendix). The total number of eggs for each cross is shown above the bars.

Bidirectional cytoplasmic incompatibility between mosquitoes infected with *w*AlbB and *w*Pip was nearly complete in both directions (Fig 1, Fig D and Tables C and D in S1 Appendix). In crosses between Palmyra-*w*AlbB males (“DQB”) with Oahu-*w*Pip females, only 0.93% of 18,457 eggs from 118 females hatched (99.07% were inviable), and in crosses with Maui-*w*Pip females, only 1.54% of 13,898 eggs from 77 females hatched (98.46% were inviable) (Fig 1, Fig D and Table C in S1 Appendix). Similarly, when Palmyra-*w*AlbB females were mated with Oahu-*w*Pip males only 0.03% of 10,496 eggs from 107 females hatched (99.97% inviable), and in crossed with Maui-wPip males only 0.05% of 14,706 eggs from 95 females hatched (99.95% inviable) (Fig 1, Fig D and Table D in S1 Appendix). In contrast, 76–88% of eggs hatched in matings between mosquitoes with the same *Wolbachia* type (Fig 1 [MP, OA, OP, and PA] and Fig D and Table E in S1 Appendix); the fraction not hatching was much higher than in mixed *Wolbachia* crosses (generalized linear model with a binomial distribution: Fraction not hatching = 4.97–6.52 (SE 0.052) * Mixed; Z = -124.3, P < 2 x10^-16^, Table F in S1 Appendix). *w*AlbB *Wolbachia* infection had minor effects on fecundity and adult female survival (Results, Figs D and E and Tables G and H in S1 Appendix).

### Vector competence measurements

We infected fourteen canaries with *P*. *relictum* GRW4, which had peak parasitemias averaging 1.22% (range 0.04%–4.7%; note that we didn’t sample birds every day and could have missed individual peak parasitemias). Peak parasitemia increased with passage number (Log(peak parasitemia) = -6.81+ 0.465 (SE 0.19) * Passage number; P = 0.032; N = 13; R^2^ = 35.3%), but was unrelated to the dose injected (# of parasites), or isolate (dose coef.: -0.39, SE 4.02, N = 13, P = 0.925; isolate coef., Pu’u Wa’awa’a vs Captain Cook: -0.042, SE 0.68, N = 13, P = 0.952, in separate models with passage number). Over the course of infection, birds had daily parasitemias of 0.016% to 4.7% and we used parasitemias between 0.028% and 2.49% to measure vector competence of *C*. *quinquefasciatus* mosquitoes (Fig F in S1 Appendix).

**Fig 2:**
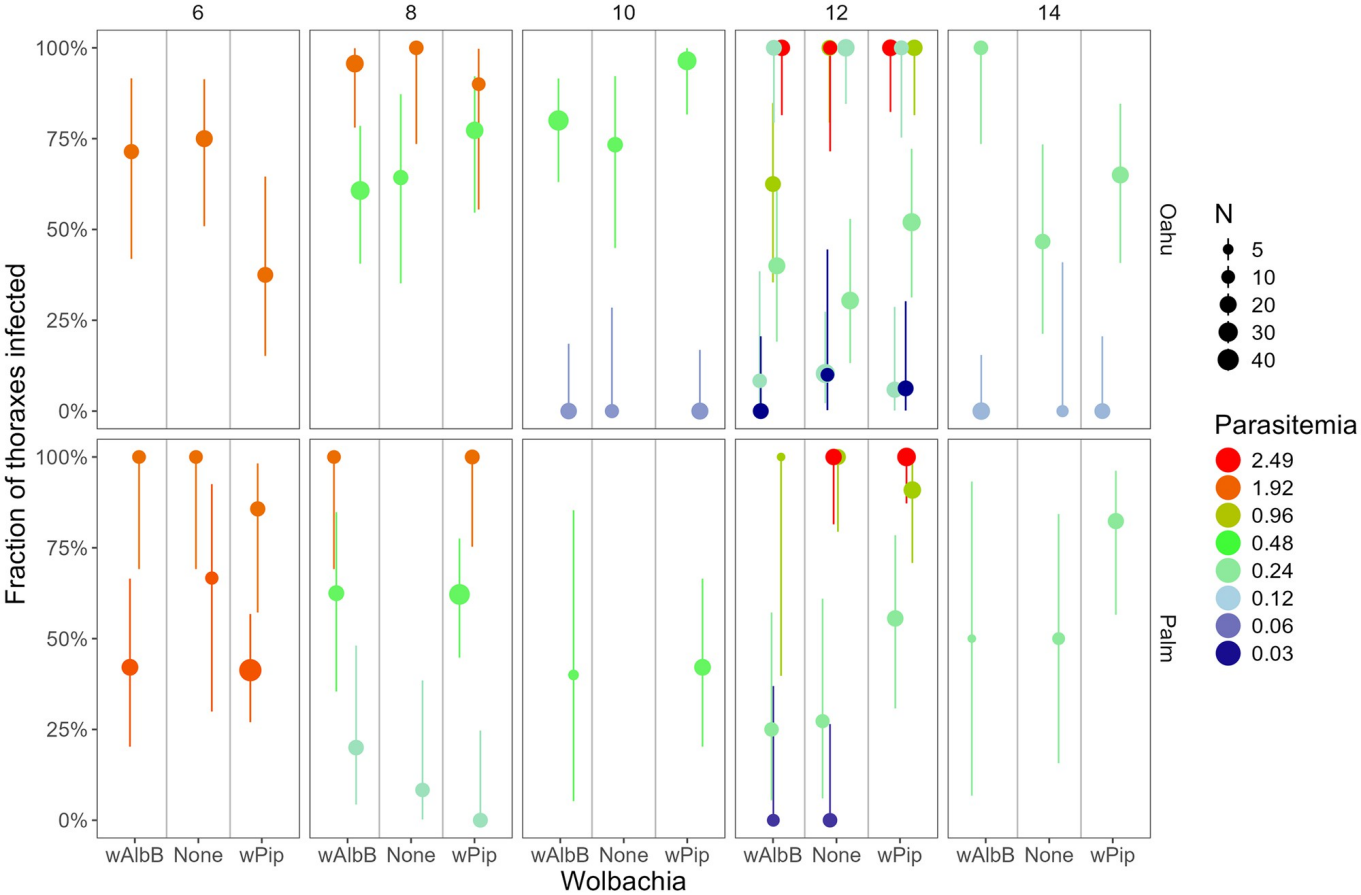
Fraction of thoraxes infected (and binomial 95% CI) plotted against *Wolbachia* type in the mosquitoes (*w*AlbB, *w*Pip, or None). The color shows the parasitemia (percent of red blood cells infected) of the bird the mosquitoes fed upon (on a log_2_ scale), the panel rows show the mosquito strain (Oahu or Palmyra), the panel columns show the days post-feeding when the mosquitoes were dissected (6–14 days), and the size of the points shows the sample size for each point (mean 16, range 4–47). The fitted model (Table I in S1 Appendix) indicates there are no consistent differences among *Wolbachia* types (points of the same color in each panel are, on average, at the same height). Points have been slightly jittered along the x-axis to aid in visualization. Note that in a small number of experiments one of the three *Wolbachia* groups had insufficient mosquitoes that successfully fed and this group is not shown (e.g. bottom middle panel, Palm-None on day 10).

**Fig 3:**
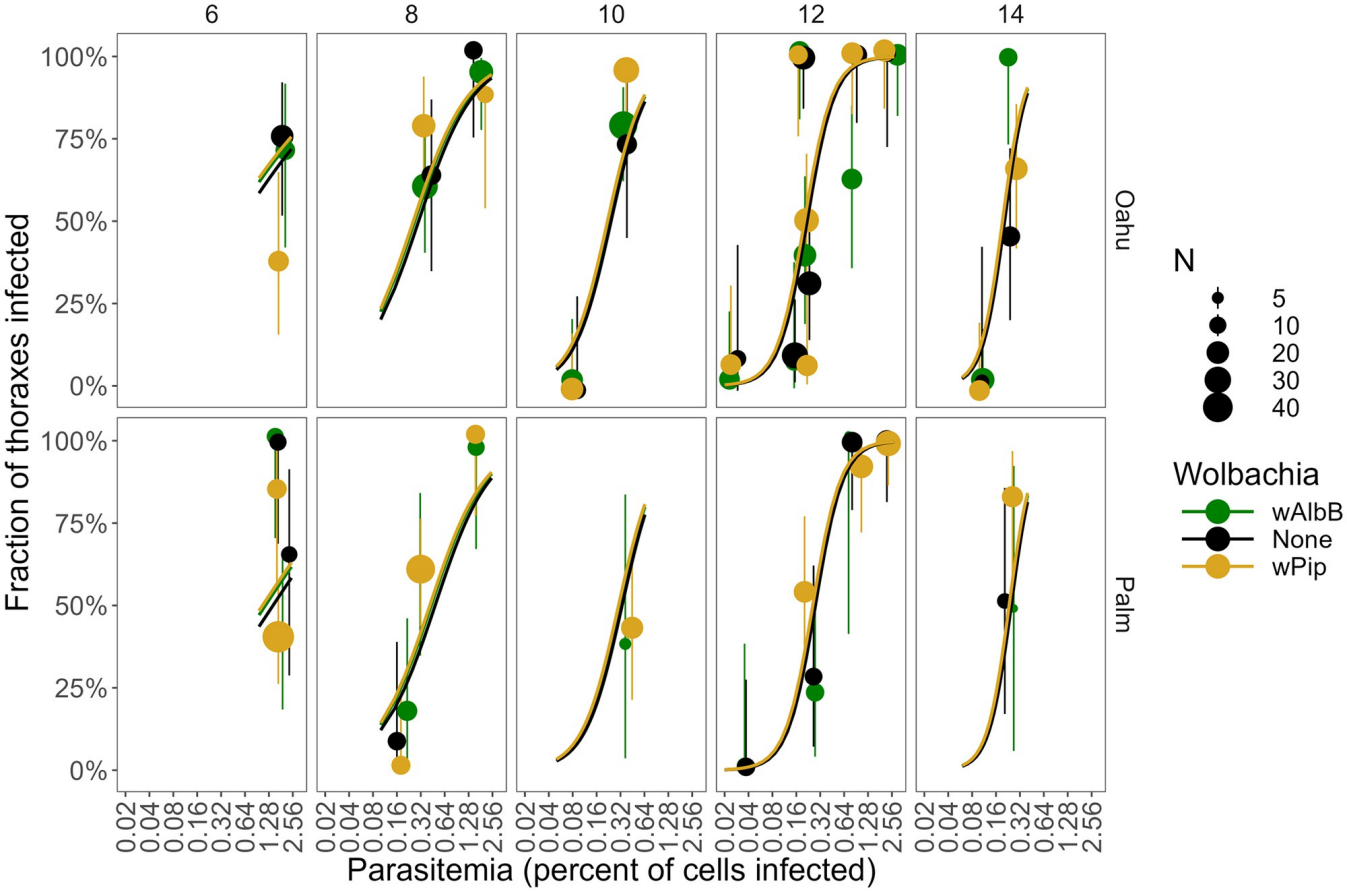
Fraction of thoraxes infected (and binomial 95% CI) plotted against the parasitemia (percent of red blood cells infected) of the bird the mosquitoes fed upon (on a log_2_ scale). This is the same data as in Fig 2, plotted with a different x-axis. Color shows the *Wolbachia* type in the mosquitoes (*w*AlbB, *w*Pip, or None), the panel rows show the mosquito strain (Oahu or Palmyra), the panel columns show the days post-feeding when the mosquitoes were dissected (6–14 days), and the size of the points shows the sample size (mean 16, range 4–47). The lines show the fitted model for each of the three *Wolbachia* types, which are on top of each other and difficult to distinguish because there was no measurable differences among *Wolbachia* types (Table I in S1 Appendix). Points have been slightly jittered along the x-axis to aid in visualization.

We fed 68 batches (mean N = 57; SD = 17.9; range 20–130) of *C*. *quinquefasciatus* mosquitoes on these infected canaries (Results: Feeding success; Fig G and Table B in S1 Appendix). We tested the 1176 fed mosquitoes from these 68 batches in 73 groups for abdomen and thorax infection, by qPCR (mean N = 16/group; SD = 7.5; range 4–47). Despite this enormous number of groups, there was no evidence for differences in thorax infection prevalence among *Wolbachia* strains (*w*AlbB, native *w*Pip, or None) (compare the height of points with the same color in each panel of Fig 2 and height of different colored lines in Fig 3; Tables I and J in S1 Appendix).

However, the fraction of mosquitoes with thorax infections increased sharply with parasitemia and days since feeding, and the slope of parasitemia increased over time (Fig 3 and Tables I and J in S1 Appendix). Thorax infection prevalence was slightly higher in the Oahu strain than in the Palmyra strain (Fig H and Tables I and J in S1 Appendix), and highest in the Field strain (Fig H and Tables I and J in S1 Appendix). The Results were qualitatively identical when we analyzed each *Wolbachia*-mosquito strain pair separately (Fig I and Table J in S1 Appendix).

**Fig 4:**
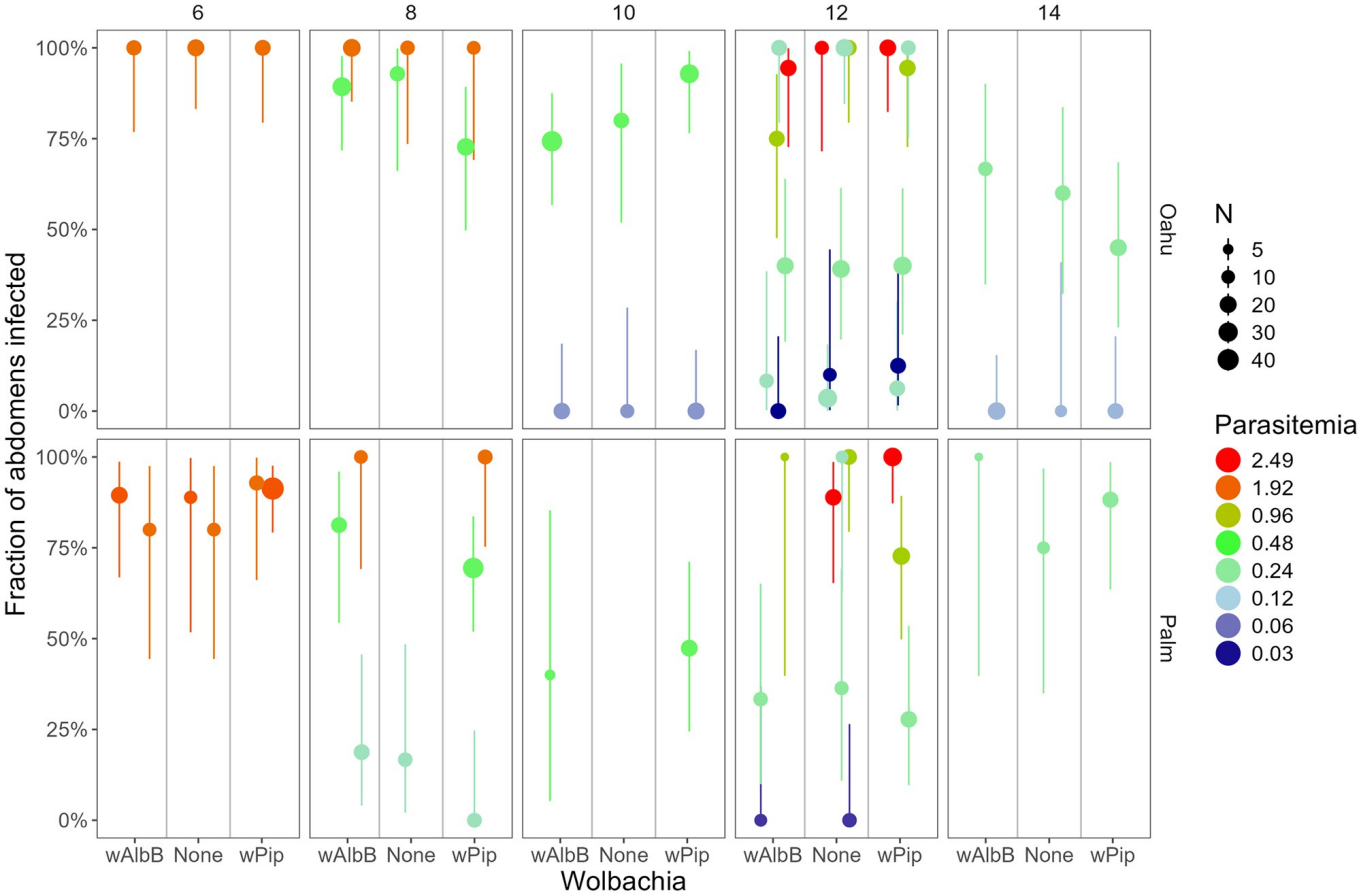
Fraction of abdomens infected (and 95% CI) plotted against *Wolbachia* type in the mosquitoes (*w*AlbB, *w*Pip, or None). The color shows the parasitemia (percent of red blood cells infected) of the bird the mosquitoes fed upon (on a log_2_ scale), the panel rows show the mosquito strain (Oahu or Palmyra), the panel columns show the days post-feeding when the mosquitoes were dissected (6–14 days), and the size of the points shows the sample size (mean 16, range 4–47). The fitted model (Table K in S1 Appendix) indicates there are no consistent differences among *Wolbachia* types (points of the same color in each panel are, on average, at the same height). Points have been slightly jittered along the x-axis to aid in visualization.

Abdomen infection prevalence was higher than thorax infection prevalence, but patterns were similar, with there being no detectable effect of *Wolbachia* type, and infection prevalence increased with parasitemia, and differed between the mosquito strains (Fig 4, Fig J and Table K in S1 Appendix). Abdomen infection prevalence increased faster with days since infection for the Palmyra and Field strains than for the Oahu strain, which resulted in differences between the mosquito strains changing with days since feeding (prevalence was higher in Oahu mosquitoes 6–10 days after infection but was higher in the Palmyra and Field strains 12–14 days after infection; Fig J and Table K in S1 Appendix).

**Fig 5:**
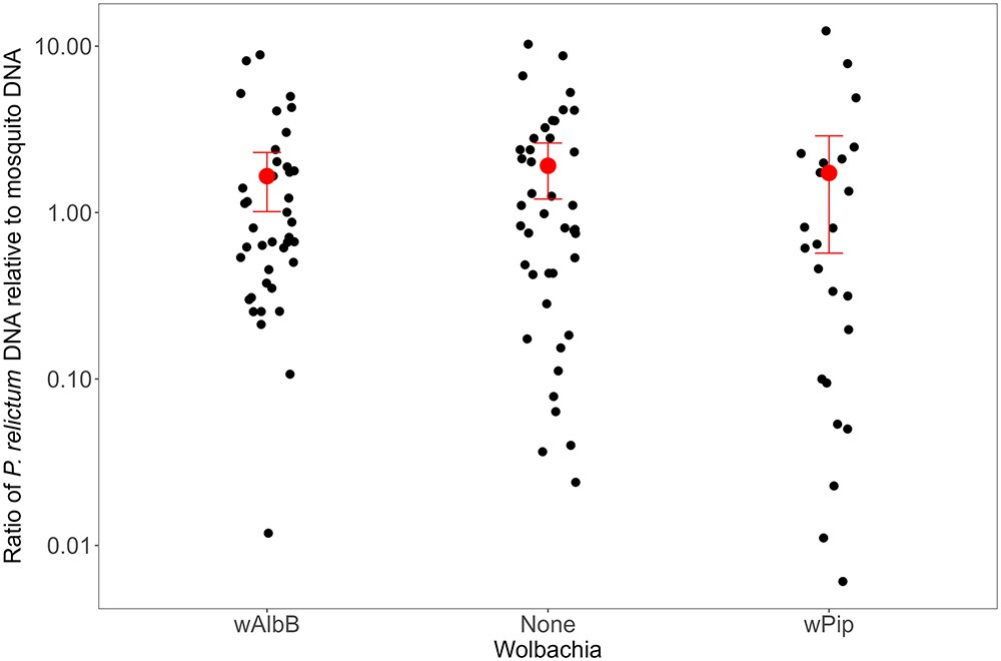
*Wolbachia* strain and salivary gland infection. *P*. *relictum* DNA to mosquito DNA measured by ddPCR, on a log_10_ scale, in salivary glands from 106 Oahu mosquitoes infected *w*AlbB, *w*Pip, or no *Wolbachia* infection. Red points show mean and 95% CI. There was no difference among *Wolbachia* types (robust F = 1.16, df = 2, P = 0.32).

We tested salivary glands from 106 Oahu mosquitoes for *P*. *relictum* DNA by ddPCR that had fed on a canary with a parasitemia of 0.12% 14 days earlier. Salivary glands from all 106 mosquitoes tested positive for *P*. *relictum* DNA, and there were no differences in the amount of *P*. *relictum* DNA among the three *Wolbachia* types (Fig 5).

## Discussion

We successfully transinfected the *w*AlbB *Wolbachia* strain into *C*. *quinquefasciatus* mosquitoes originating from Palmyra Atoll after clearing them of their natural *w*Pip *Wolbachia* infection. The resultant Palmyra-*w*AlbB line of mosquitoes exhibited complete transmission of *w*AlbB across generations, and almost 100% bidirectional CI with mosquitoes from Hawaii (Maui and Oahu) infected with *w*Pip. We then created another *w*AlbB-infected line by backcrossing the Palmyra-*w*AlbB into an Oahu strain of *C*. *quinquefasciatus* that we cleared of natural *w*Pip infection. The creation of these two lines of *w*AlbB-infected *C*. *quinquefasciatus* mosquitoes makes population suppression of *C*. *quinquefasciatus* via widespread release of *w*AlbB-infected males possible both in Hawaii and elsewhere. The bidirectional CI we observed between *w*AlbB and *w*Pip mosquitoes would result in any accidentally released *w*AlbB-infected females having inviable offspring if they mate with native *w*Pip males (but not if they mate with *w*AlbB-infected males).

We then examined the effect of *w*AlbB, *w*Pip or no *Wolbachia* on vector competence in two mosquito strains, Palmyra and Oahu, for *P*. *relictum* GRW4, the only lineage of avian malaria detected in Hawaii [2]. We found infection with *Wolbachia* strains *w*AlbB and *w*Pip had no detectable effect on thorax or abdomen infection of *C*. *quinquefasciatus* mosquitoes for avian malaria *P*. *relictum* GRW4 in either the Oahu or Palmyra mosquito strains. We also found no difference among *Wolbachia* groups in the amount of *P*. *relictum* DNA in salivary glands in a subset of mosquitoes from the Oahu strain. This is the first study, to our knowledge, to experimentally assess the impact of both natural infections and stable transinfections of *Wolbachia* on malaria vector competence in the same natural mosquito-pathogen system (but see [31] for an observational study of natural *Wolbachia* infection and *Plasmodium* prevalence in *Anopheles coluzzii*). Taken together these results indicate that the purposeful or accidental introduction of the *w*AlbB strain of *Wolbachia* to mosquito populations in Hawaii would likely neither help nor hurt conservation efforts to reduce transmission of avian malaria [32,33].

Our results differ from two previous studies that found that *Wolbachia* altered *Plasmodium* prevalence in malaria vectors. In *C*. *quinquefasciatus*, natural *w*Pip infections increased vector competence for *P*. *relictum* lineage SGS1 compared to those cleared of *Wolbachia* infection [21]. In contrast, in *Anopheles stephensi*, transinfected *w*AlbB reduced midgut infection prevalence of *Plasmodium falciparum* [34]. The conflicting results between these two studies suggests that the impacts of *Wolbachia* on *Plasmodium* infection are not uniform, which is consistent with the highly variable effects of transinfected *Wolbachia* on other pathogens in other vectors [9]. We had much larger sample sizes than all past studies combined, and examined seven lines of mosquitoes, including two inbred mosquito strains with two strains of *Wolbachia* or no *Wolbachia* and one Field mosquito strain, suggesting that a lack of an effect of *Wolbachia* on *Plasmodium* vector competence was not due to a lack of power in our study.

In contrast to the lack of differences among *Wolbachia* lines, we found moderate differences among mosquito strains in vector competence, with Field mosquitoes having the highest vector competence, followed by the inbred Oahu strain and then the inbred Palmyra strain. The differences in vector competence among mosquito strains indicates that we had ample power to detect differences in vector competence. They also underline the extensive variability in vector competence among populations of the same mosquito species, which is common for many vector-pathogen pairs [35,36].

We found a relatively steep relationship between parasitemia and both thorax and abdomen infection, and the relationship became steeper with time since feeding. Twelve days after feeding the relationship was very steep, with parasitemias up to 0.1% leading to relatively few disseminated thorax infections (<15.0%), whereas parasitemias only three-fold higher (0.3%) led to most (73.0%) mosquitoes having thorax infections. In contrast, after only eight days after feeding, a much higher 10-fold range of parasitemias (0.06% to 0.6%) was required to produce a similar range in thorax infection. The steep relationship between host parasitemia and thorax infection prevalence made it more challenging to determine the appropriate day to feed mosquitoes on a canary and which day to dissect and test mosquitoes to obtain an intermediate level of infection. In many of our feedings most or almost none of the mosquitoes in all groups had thorax infections (Figs 2 and 3, H and I in S1 Appendix). This steep relationship, if it is also present in wild mosquitoes, would lead to highly heterogeneous infectiousness among birds (and bird species), with birds infecting nearly all or almost none of the mosquitoes that fed on them depending on whether their parasitemia was above or below a relatively narrow threshold parasitemia (e.g. ~0.1%-0.3% for day 12 post feeding). Our data from the Field mosquito strain was limited, with only one day post feeding (day 12) where we had fed mosquitoes on canaries with a range of parasitemias (four parasitemias, ranging from 0.028% to 1.225%; Fig K in S1 Appendix). Analysis of just this (small) dataset of wild G0 mosquitoes produced a much less steep relationship (Fig K in S1 Appendix), suggesting the steep relationship we observed for the dataset composed of mosquitoes from two inbred lines (Oahu and Palmyra), may be a result of limited genetic variation.

In summary, the rapid decline of many species of Hawaiian birds over the last several decades, due to a climate-change driven increase in malaria transmission at higher elevations, requires urgent action to prevent extinction [1,5,7,37]. We created two lines of *w*AlbB-infected *C*. *quinquefasciatus* mosquitoes with high CI with Hawaiian *C*. *quinquefasciatus* infected with *w*Pip, which could be used to suppress mosquito populations via widespread release of *w*AlbB-infected males. We examined whether the transinfected *Wolbachia* strain *w*AlbB increased or decreased vector competence of these two lines for *P*. *relictum* GRW4. We found no effect of *Wolbachia* infection on thorax, abdomen, or salivary gland infection, suggesting that replacement of the current *Wolbachia* in Hawaiian mosquitoes (*w*Pip) with the *w*AlbB strain would have little impact on their susceptibility to infection and ability to transmit this parasite. Furthermore, vector competence in one population of mosquitoes collected directly from the field had much higher infection rates than the two transinfected lines. This suggests that accidental releases of small numbers of female mosquitoes with *Wolbachia* strain *w*AlbB with the large numbers of male mosquitoes with *Wolbachia* strain *w*AlbB that is planned for 2024 [32,33] is unlikely to alter the transmission of malaria beyond the effect of the *w*AlbB-infected males greatly reducing mosquito populations.

## Supporting information

S1 Appendix Supplemental text, tables and figures. **Supplemental text includes Methods: Mosquito marking, Methods**: Mosquito marking, *Plasmodium relictum* qPCR and ddPCR, Methods: Salivary Gland Infection, Results: Feeding success, Results: Fecundity and Adult Female Survival; Tables A K, Figs A- K.
(DOCX)
