# Supplementary material for: Transinfection of Wolbachia wAlbB into Culex quinquefasciatus mosquitoes does not alter vector competence for Hawaiian avian malaria (Plasmodium relictum GRW4)
Source: PLoS Pathog. 2024 Aug 5;20(8):e1012052. doi: 10.1371/journal.ppat.1012052 (PMC11326542; doi:10.1371/journal.ppat.1012052)
Supplement: S1 Appendix — Supplemental text includes Methods: Mosquito marking, Methods: Mosquito marking, Plasmodium relictum qPCR and ddPCR, Methods: Salivary Gland Infection, Results: Feeding success, Results: Fecundity and Adult Female Survival; Tables A K, Figs A- K. (DOCX) [file ppat.1012052.s001.docx]

**Supplemental text for**

**Transinfection of *Wolbachia w*AlbB into *Culex quinquefasciatus* mosquitoes does not alter vector competence for Hawaiian avian malaria (*Plasmodium relictum* GRW4)**

A. Marm Kilpatrick^1,^*, Christa M. Seidl^1^, Isaiah J. Ipsaro^1^, Chris E. Garrison^1^, Giulia Fabbri^2^, Paul I. Howell^2^, Austin G. McGowan^2^, Bradley J. White^2^, Sara N. Mitchell^2^

^1^Department of Ecology and Evolutionary Biology, University of California, Santa Cruz, California, USA

^2^Verily Life Sciences, South San Francisco, California, USA

*[akilpatr@ucsc.edu](mailto:akilpatr@ucsc.edu)

**Methods**

Mosquito Marking

In order to differentiate between mosquito lines (including *Wolbachia* strains) during the feeding assay, mosquito lines were randomly chosen to either be unmarked or sprayed with a green or red fluorescent marker (SmartWater CSI LLC, GBR) (Fig A). The two markers used were Cartax-DP Fluorescent Marker (green) and Invisible Red S marker (red), which were combined with a polymer to ensure adherence to the mosquitoes [1]. For the green marker, we mixed Cartax-DP Fluorescent Marker with Mowilith LDM 7709 Polymer *in house* using methods modified from [1]. Briefly, 60 µL of Cartax-DP Fluorescent Marker (0.5% sodium lauryl sulfate [SLS] as dispersal agent) was combined with 12 µL of Mowilith polymer in 3,928 µL of water to produce a 1.5% dye solution with a dye:polymer ratio of 5:1. To aid visualization of the red marker, we used a premixed dye-polymer also available from SmartWater (SmartTrace, SmartWater Forensic Marker Solution), which is a 0.5% Invisible Red S dye solution containing a Invisible Red SMarker:Mowilith LDM 7709 Polymer ratio of 1:1 (0.5% SLS). The fluorescent mixtures (*in house* or premixed) were then vortexed for 15 to 30 seconds and added to a nebulizer (ASOMI Mesh Nebulizer) reservoir. The nebulizer mouthpiece was connected to a plastic container containing 150 female mosquitoes and turned to the highest setting for thirty seconds. During spraying the container was tapped lightly to ensure mosquito movement for maximum coverage. The spray was allowed to dry for two hours and a small subset of mosquitoes were checked for fluorescent marking under a UV light to confirm proper application. We randomly rotated the marking of mosquito strains (red, green or no spray) for each feeding experiment.

The accuracy of this marking method was tested by molecularly identifying 95 mosquitoes that we had identified using the color marking (20 Palmyra-None marked red; 16 Palmyra-*w*AlbB marked green; 20 Oahu-None marked red; 32 Oahu-*w*Pip (unmarked); and 7 Oahu-*w*AlbB marked green). We used ddPCR to examine the presence/absence of *w*Pip or wAlbB *Wolbachia* (see [2]) with wPip *WSP* (ID WP0937) (F 5’ GCTGGTGCTCGTTATTTTGG 3’, R 5’ ACAGCGCTGTAAAGGACATT 3’, probe 5’ FAM AAGAAGCAGTATCAGCTACTAAAGAGA ZEN/3’ IBFQ). We used the RPL5 *C. quinquefasciatus* housekeeping gene as a positive ddPCR control. All but one mosquito (94/95) was correctly identified using the marking colors: one unmarked Oahu-*w*Pip mosquito was mis-identified as a red marked Oahu-None.

*Plasmodium relictum* qPCR and ddPCR

We extracted DNA from all mosquito samples for qPCR and ddPCR analysis using the Chemagic 360 DNA (PerkinElmer) extraction system (see [2] for methods), and analyzed for the GRW4 lineage of *P. relictum*. For real-time qPCR, the following oligos were used: Forward primer - 5’ ATTAGCAGAACAAAGAAACTTAACA 3’; Reverse primer - 5’ CATAGAATGAACATATAAACCAG 3’ [3]; and Probe - 5’ GCTTTTGGTGCAAGAGAGTATTCAGT 3’ (Whitaker, Kelsey 2022). The qPCR reaction mixture and set up of thermocycler profile follow the methods developed in [4]. Samples were considered positive for *P. relictum* GRW4 if they crossed the threshold at or before 40 cycles.

We diluted a subset of extracted DNA from salivary glands 1:20 with water and then analyzed it using a QX200 AutoDG Droplet Digital PCR (ddPCR) System (Bio-Rad, Laboratories). The ddPCR is a multiplexed reaction targeting both the *P. relictum* *GRW4* gene (Forward: 5’ AAATGAGTTTCTGGGGTGCT 3’, Reverse: 5’ TGGGTCACTTACAAGATATCCAC 3’, Probe: 5’ FAM CACATATCCATGAAACTAGTCCAGG3’) and the *Culex quinquefasciatus* RPL5 housekeeping gene (as detailed previously). The housekeeping gene was used to ensure quality of extracted DNA and normalize titer levels between mosquito samples. Thermal cycling conditions were 95°C for 10 minutes, followed by 40 cycles of 95°C for 30 second denaturing step and an annealing step at 55°C for 30 seconds. This was followed by a final extension at 98°C for 10 minutes. Analysis was performed using the QuantaSoft Analysis Pro Software (Bio-Rad).

Salivary Gland Infection

We dissected one or both salivary lobes from each mosquito and washed the lobe(s) three times in phosphate buffer solution to ensure that no stray tissues were still attached to the glands. We placed glands into a deep well plate with 200uL of Lysis buffer and then homogenized them with two 3mm Grinding Balls (OPS Diagnostics) at 900 RPM for two minutes. We placed the plate into a VWR Ultrasonic Cleaner and sonicated it at 35kHz for ten minutes to ensure salivary tissues were completely lysed. We then extracted DNA using the Chemagic 360 DNA (PerkinElmer) extraction system and ran DNA on the QX200 AutoDG Droplet Digital PCR System (Supplemental Information, Methods*: Plasmodium relictum* qPCR and ddPCR).

**Results**

Feeding success

Feeding success, or the fraction of mosquitoes that obtained a blood meal, varied both among mosquito strain and *Wolbachia* type (Fig G; Table B). Oahu mosquitoes were more successful at feeding than the Palmyra strain and all three Oahu *Wolbachia* groups fed similarly (Fig G; Table B). Palmyra mosquitoes with *Wolbachia* *w*Pip had higher feeding success than Palmyra mosquitoes with the wAlbB *Wolbachia* strain or those cleared of *Wolbachia* (Fig G; Table B). In addition, somewhat surprisingly, mosquitoes sprayed red had significantly lower feeding success than those sprayed green or with no spray, which did not differ significantly from each other (Fig G; Table B). This difference may be due to a higher polymer concentration in the red premix dye vs the green markers, or some other factor.

Fecundity and Adult Female Survival

Fecundity was higher for Oahu mosquitoes than Maui or Palmyra mosquitoes and was lower for crosses where males were infected with *w*Pip compared to *w*AlbB (Fig D, Table F). Similarly, adult female survival for 51 groups (a strain-*Wolbachia* type was a group) of mosquitoes (1535 total mosquitoes) in the eleven days after blood feeding was higher for Oahu mosquitoes than the Palmyra strain and was slightly lower for females infected with *w*AlbB than *w*Pip. However, the differences among *Wolbachia* types was small, and assessment of survival was limited to the eleven days after blood feeding at one temperature and may have been influenced by the availability of sugar water, and the handling, marking, and transport of mosquitoes (Fig E, Table G).

**References**

1. Faiman R, Krajacich BJ, Graber L, Dao A, Yaro AS, Yossi O, et al. A novel fluorescence and DNA combination for versatile, long-term marking of mosquitoes. Methods Ecol Evol. 2021;12: 1008–1016. doi:10.1111/2041-210X.13592

2. Crawford JE, Clarke DW, Criswell V, Desnoyer M, Cornel D, Deegan B, et al. Efficient production of male Wolbachia-infected Aedes aegypti mosquitoes enables large-scale suppression of wild populations. Nat Biotechnol. 2020;38: 482–492. doi:10.1038/s41587-020-0471-x

3. Zehtindjiev P, Ilieva M, Westerdahl H, Hansson B, Valkiūnas G, Bensch S. Dynamics of parasitemia of malaria parasites in a naturally and experimentally infected migratory songbird, the great reed warbler Acrocephalus arundinaceus. Exp Parasitol. 2008;119: 99–110. doi:10.1016/j.exppara.2007.12.018

4. Videvall E, Paxton KL, Campana MG, Cassin-Sackett L, Atkinson CT, Fleischer RC. Transcriptome assembly and differential gene expression of the invasive avian malaria parasite Plasmodium relictum in Hawaiʻi. Ecol Evol. 2021;11: 4935–4944. doi:10.1002/ece3.7401

5. Dumas E, Atyame CM, Milesi P, Fonseca DM, Shaikevich EV, Unal S, et al. Population structure of Wolbachia and cytoplasmic introgression in a complex of mosquito species. BMC Evol Biol. 2013;13: 181. doi:10.1186/1471-2148-13-181

**Supplemental Tables and Figs**

**Table A. *Culex quinquefasciatus* mosquito lines used for three types of experiments (VC - vector competence for avian malaria (*Plasmodium relictum* GRW4), TW - transmission of *Wolbachia* strain *w*AlbB, CI - Cytoplasmic incompatibility).** Mosquito lines are referred to using their mosquito strain and *Wolbachia* strain joined with a dash (e.g. Palm-*w*AlbB). *w*Pip clade assignment was performed using molecular markers Ank2 and PK1 [5].

| **Line** | **Origin** | **Wolbachia** | **Experiments** | **Notes** |
| --- | --- | --- | --- | --- |
| Palm-*w*Pip | Palmyra Atoll, USA | *w*Pip (clade 3) | VC | Colony in generation since 2018 |
| Palm-None | Palmyra Atoll, USA | None (*w*Pip cleared) | VC | Created from Palmyra-*w*Pip |
| Palm-*w*AlbB | Palmyra Atoll, USA | *w*AlbB (KLP) transinfected | VC, TW, CI | Original *w*AlbB line; referred to as DQB3 in government documents |
| Oahu-*w*Pip | Oahu, USA | *w*Pip (clade 5) | VC, CI | Colony in generation since 2020 |
| Oahu-None | Oahu, USA | None (*w*Pip cleared) | VC | Created from Oahu-*w*Pip |
| Oahu-*w*AlbB | Oahu, USA | *w*AlbB (KLP) infected via backcross | VC | Generated by crossing males from line Oahu-None with females from line Palm-*w*AlbB for 7 generations |
| Maui-*w*Pip | Maui, USA | *w*Pip | CI | Colony in generation since 2020 |
| Field | Hawaii, USA | *w*Pip | VC | G0 wild mosquitoes, collected 2023 |

**Table B. Statistical analysis of feeding success shown in Fig G.** The generalized linear mixed effects model with a binomial distribution and a logit link had mosquito strain (reference level: Oahu), *Wolbachia* strain (reference level: *w*AlbB), spray color, a two-way interaction between mosquito strain and *Wolbachia* strain, and a random effect for bird ID as predictors. There was significant variation among spray colors (𝛘^2^ = 28.5, df = 2, P = 6.50x10^-07^), mosquito strains (𝛘^2^ = 7.9, df = 1, P = 0.0049), *Wolbachia* types (𝛘^2^ = 20.9, df = 2, P = 2.95x10^-05^), and the two-way interaction between mosquito strain and *Wolbachia* strain was also significant (𝛘^2^ = 31.9, df = 2, P = 1.19x10^-07^). The bird ID random effect variance was 0.50.

| **Predictor** | **Estimate** | **SE** | **z value** | **P-value** |
| --- | --- | --- | --- | --- |
| **(Intercept)** | 0.42 | 0.24 | 1.73 | 0.084 |
| **Spray(None)** | -0.031 | 0.085 | -0.37 | 0.71 |
| **Spray(Red)** | -0.40 | 0.084 | -4.78 | 1.72x10^-6^ |
| **strain(Palm)** | -0.66 | 0.12 | -5.59 | 2.26x10^-8^ |
| ***Wolbachia*(None)** | -0.065 | 0.11 | -0.58 | 0.56 |
| ***Wolbachia*(*w*Pip)** | -0.036 | 0.11 | -0.32 | 0.75 |
| **strain(Palm)**Wolbachia*(None)** | 0.47 | 0.16 | 2.84 | 0.0045 |
| **strain(Palm)**Wolbachia*(*w*Pip)** | 0.94 | 0.17 | 5.65 | 1.63x10^-8^ |

**Table C. Cytoplasmic Incompatibility (CI) results for the male Palmyra-*w*AlbB line for three biological replicates.** Each replicate consisted of 150 males of the Palmyra-*w*AlbB line mated to 100 females infected with *Wolbachia* *w*Pip from Oahu or Maui. Eggs were assessed for hatching either from individual females (Isofemales) or from eggs laid *en masse* in cages (Mass Cages).

| **Rep.** | **Male** | **Female** | **# females** | **# eggs** | **# larvae** | **% CI** |
| --- | --- | --- | --- | --- | --- | --- |
| 1 | Palmyra-*w*AlbB | Oahu-*w*Pip | 41 | 7334 | 90 | 98.77% |
| 2 | Palmyra-*w*AlbB | Oahu-*w*Pip | 45 | 5062 | 35 | 99.31% |
| 3 | Palmyra-*w*AlbB | Oahu-*w*Pip | 32 | 7131 | 55 | 99.23% |
|  |  | **Oahu total** | **118** | **19527** | **180** | **99.08%** |
| 1 | Palmyra-*w*AlbB | Maui-*w*Pip | 43 | 7914 | 72 | 99.09% |
| 2 | Palmyra-*w*AlbB | Maui-*w*Pip | en masse | 786 | 26 | 96.69% |
| 3 | Palmyra-*w*AlbB | Maui-*w*Pip | 34 | 5198 | 117 | 97.75% |
|  |  | **Maui total** | **77** | **13898** | **215** | **98.45%** |
|  |  | **Totals** | **195** | **33425** | **395** | **98.82%** |

**Table D. Cytoplasmic Incompatibility results for female Palmyra-*w*AlbB line with males of two *w*Pip lines for three biological replicates.** Each replicate consists of 150 males from Oahu or Maui infected with *w*Pip mated to 100 Palmyra-*w*AlbB females. Eggs were assessed for hatching from individual females (Isofemales).

| **Rep.** | **Male** | **Female** | **# females** | **# eggs** | **# larvae** | **% CI** |
| --- | --- | --- | --- | --- | --- | --- |
| 1 | Oahu-*w*Pip | Palmyra-*w*AlbB | 45 | 7251 | 0 | 100.00% |
| 2 | Oahu-*w*Pip | Palmyra-*w*AlbB | 17 | 1401 | 0 | 100.00% |
| 3 | Oahu-*w*Pip | Palmyra-*w*AlbB | 45 | 6837 | 5 | 99.93% |
|  |  | **Oahu total** | **107** | **15489** | **5** | **99.97%** |
| 1 | Maui-*w*Pip | Palmyra-*w*AlbB | 26 | 4061 | 7 | 99.83% |
| 2 | Maui-*w*Pip | Palmyra-*w*AlbB | 40 | 7002 | 6 | 99.91% |
| 3 | Maui-*w*Pip | Palmyra-*w*AlbB | 29 | 3643 | 0 | 100.00% |
|  |  | **Maui total** | **95** | **14706** | **13** | **99.91%** |
|  |  | **Total** | **202** | **30195** | **18** | **99.94%** |

**Table E. Egg viability results for four control matings with the same *Wolbachia* type in males and females, in two different genetic backgrounds for each *Wolbachia* type (*w*AlbB and *w*Pip).**

| **Abbrev.**  **Fig 1** | **Male** | **Female** | **# females** | **# eggs** | **# larvae** | **% CI** |
| --- | --- | --- | --- | --- | --- | --- |
| MP | Maui-*w*Pip | Maui-*w*Pip | 40 | 4401 | 517 | 11.75% |
| OA | Oahu-*w*AlbB | Oahu-*w*AlbB | 29 | 5805 | 1397 | 24.07% |
| OP | Oahu-*w*Pip | Oahu-*w*Pip | 96 | 16269 | 2643 | 16.25% |
| PA | Palmyra-*w*AlbB | Palmyra-*w*AlbB | 30 | 6345 | 1194 | 18.82% |

**Table F. Statistical analysis of egg inviability shown in Fig 1.** The generalized linear model with a binomial distribution and a logit link had mosquito strain (reference level: Maui), and cross (reference level: Maui, Male *w*Pip-Female *w*Pip). Egg inviability**/**CI differed among strains (𝛘^2^ = 105, df = 2, P < 2x10^-16^) and crosses (𝛘^2^ = 71,714, df = 3, P < 2x10^-16^). The coefficients for each Cross or Female strain indicate the difference between that cross or strain and the reference level.

| **Predictor** | **Estimate** | **SE** | **z value** | **P-value** |
| --- | --- | --- | --- | --- |
| **Intercept (Maui, Male *w*Pip*-*Female *w*Pip)** | -2.00 | 0.042 | -47.24 | 2x10^-16^ |
| **Cross (Male *w*AlbB- Female *w*AlbB)** | 0.50 | 0.037 | 13.52 | 2x10^-16^ |
| **Cross (Male *w*AlbB- Female *w*Pip)** | 6.22 | 0.056 | 111.50 | 2x10^-16^ |
| **Cross (Male *w*Pip-Female *w*AlbB)** | 12.96 | 1.42 | 9.16 | 2x10^-16^ |
| **Female strain (Oahu)** | 0.35 | 0.046 | 7.62 | 2.6x10^-14^ |
| **Female strain (Palm)** | 0.050 | 0.064 | 0.79 | 0.43 |

**Table G. Statistical analysis of fecundity of *C. quinquefasciatus* mosquitoes with two *Wolbachia* types and from three genetic backgrounds in four different crosses.** The generalized linear mixed effects model with a negative binomial distribution and a log link had mosquito strain (reference level: Maui), and male and female *Wolbachia* strain (reference level: *w*AlbB). There was significant variation among female strains (𝛘^2^ = 13.2, df = 2, P = 0.0014).

| **Predictor** | **Estimate** | **SE** | **z value** | **P-value** |
| --- | --- | --- | --- | --- |
| **(Intercept)** | 5.19 | 0.086 | 60.01 | <2x10^-16^ |
| **Female strain (Oahu)** | 0.15 | 0.045 | 3.28 | 0.0011 |
| **Female strain (Palm)** | 0.026 | 0.097 | 0.27 | 0.79 |
| **Female Wolbachia (*w*Pip)** | -0.13 | 0.080 | -1.60 | 0.11 |
| **Male Wolbachia (*w*Pip)** | -0.18 | 0.038 | -4.77 | 1.85 x10^-6^ |

**Table H. Cox’s proportional hazard survival analysis of *C. quinquefasciatus* mosquitoes with *Wolbachia* (No Wolbachia was the reference level) and Strain (Oahu was the reference level).** Note that a positive coefficient indicates higher mortality (hazard) or lower survival and vice-versa.

| **Predictor** | **Estimate** | **SE** | **z value** | **P-value** |
| --- | --- | --- | --- | --- |
| ***Wolbachia* (*w*AlbB)** | 0.53 | 0.28 | 1.90 | 0.057 |
| ***Wolbachia* (*w*Pip)** | -0.010 | 0.28 | -0.036 | 0.97 |
| **Strain (Palm)** | 0.66 | 0.23 | 2.88 | 0.00404 |
| **Age at Feeding** | 0.12 | 0.049 | 2.44 | 0.01 |

**Table I. Statistical analysis of disseminated (thorax) infections shown in Figs 2, 3, and Fig H.** The generalized linear model with a binomial distribution and a logit link had log(Parasitemia), days post feeding, mosquito strain (reference level: Oahu), *Wolbachia* type (reference level: *w*AlbB), and a two-way interaction between ln(Parasitemia) and days post feeding as predictors. There was significant variation among mosquito strains (𝛘^2^ = 102.0, df = 2, P < 2x10^-16^), but not *Wolbachia* strains (𝛘^2^ = 1.15, df = 2, P = 0.56). This model fit better than a model without the two way log(Parasitemia)*days since feeding interaction (ΔAIC = 81.0), and better than models with additional two-way interactions (Parasitemia*strain ΔAIC = 2.99; Parasitemia*strain+days since feeding*strain; ΔAIC = 2.75).

| **Predictor** | **Estimate** | **SE** | **z value** | **P-value** |
| --- | --- | --- | --- | --- |
| **(Intercept)** | -2.91 | 0.46 | -6.38 | 1.72x10^-10^ |
| **ln(Parasitemia)** | -1.23 | 0.33 | -3.73 | 0.000193 |
| **Days since feeding** | 0.57 | 0.0579 | 9.84 | < 2x10^-16^ |
| **strain(Palm)** | -0.60 | 0.16 | -3.69 | 0.000227 |
| **strain(Field)** | 2.99 | 0.45 | 6.62 | 3.69x10^-11^ |
| ***Wolbachia*(None)** | -0.14 | 0.19 | -0.74 | 0.46 |
| ***Wolbachia*(*w*Pip)** | 0.052 | 0.17 | 0.30 | 0.76 |
| **ln(Parasitemia)***  **Days post feeding** | 0.31 | 0.038 | 8.12 | 4.54x10^-16^ |

**Table J. Statistical analysis of thorax infections using individual mosquito-*Wolbachia* strain pairs shown in Fig I.** The generalized linear model with a binomial distribution and a logit link had log(Parasitemia), days post feeding, mosquito strain - *Wolbachia* strain pair (reference level: Oahu-*w*AlbB), and a two-way interaction between ln(Parasitemia) and days post feeding as predictors. There was significant variation among mosquito strain - *Wolbachia* pairs (𝛘^2^ = 114.8, df = 6, P < 2x10^-16^), but not *Wolbachia* strains within mosquito strains (see coefficients and SEs below).

| **Predictor** | **Estimate** | **SE** | **z value** | **P-value** |
| --- | --- | --- | --- | --- |
| **(Intercept)** | -2.94 | 0.47 | -6.31 | 2.8x10^-10^ |
| **ln(Parasitemia)** | -1.23 | 0.33 | -3.73 | 0.00020 |
| **Days post feeding** | 0.57 | 0.058 | 9.85 | < 2x10^-16^ |
| **strain(Oahu- none)** | -0.083 | 0.24 | -0.35 | 0.73 |
| **strain(Oahu-*w*Pip)** | 0.072 | 0.23 | 0.31 | 0.76 |
| **strain(Palm-*w*AlbB)** | -0.53 | 0.28 | -1.93 | 0.053 |
| **strain(Palm-none)** | -0.79 | 0.30 | -2.60 | 0.0093 |
| **strain(Palm-*w*Pip)** | -0.52 | 0.23 | -2.25 | 0.025 |
| **strain(Field-*w*Pip)** | 3.07 | 0.46 | 6.63 | 3.45x10^-11^ |
| **ln(Parasitemia)***  **Days post feeding** | 0.31 | 0.038 | 8.12 | 4.58x10^-16^ |

**Table K.** **Statistical analysis of abdomen infections shown in Fig 4.** The generalized linear model with a binomial distribution and a logit link had ln(Parasitemia), days post feeding, mosquito strain (reference level: Oahu), *Wolbachia* strain (reference level: *w*AlbB), and a two-way interaction between days post feeding and mosquito strain as predictors. There was significant variation among mosquito strains (𝛘^2^ = 95.9, df = 2, P < 2x10^-16^), but not *Wolbachia* strains (𝛘^2^ = 1.27, df = 2, P = 0.53).

| **Predictor** | **Estimate** | **SE** | **z value** | **P-value** |
| --- | --- | --- | --- | --- |
| **(Intercept)** | 4.53 | 0.67 | 6.74 | 1.63x10^-11^ |
| **ln(Parasitemia)** | 1.84 | 0.12 | 15.13 | < 2x10^-16^ |
| **Days post feeding** | -0.15 | 0.060 | -2.57 | 0.010 |
| **strain(Palm)** | -4.50 | 0.85 | -5.31 | 1.07x10^-7^ |
| **strain(Field)** | -8.64 | 1.57 | -5.50 | 3.88x10^-8^ |
| ***Wolbachia*(None)** | 0.17 | 0.21 | 0.82 | 0.41 |
| ***Wolbachia*(*w*Pip)** | -0.044 | 0.19 | -0.23 | 0.82 |
| **Days post feeding*Palm** | 0.38 | 0.077 | 4.92 | 8.56x10^-7^ |
| **Days post feeding*Field** | 1.01 | 0.15 | 6.78 | 1.20x10^-11^ |


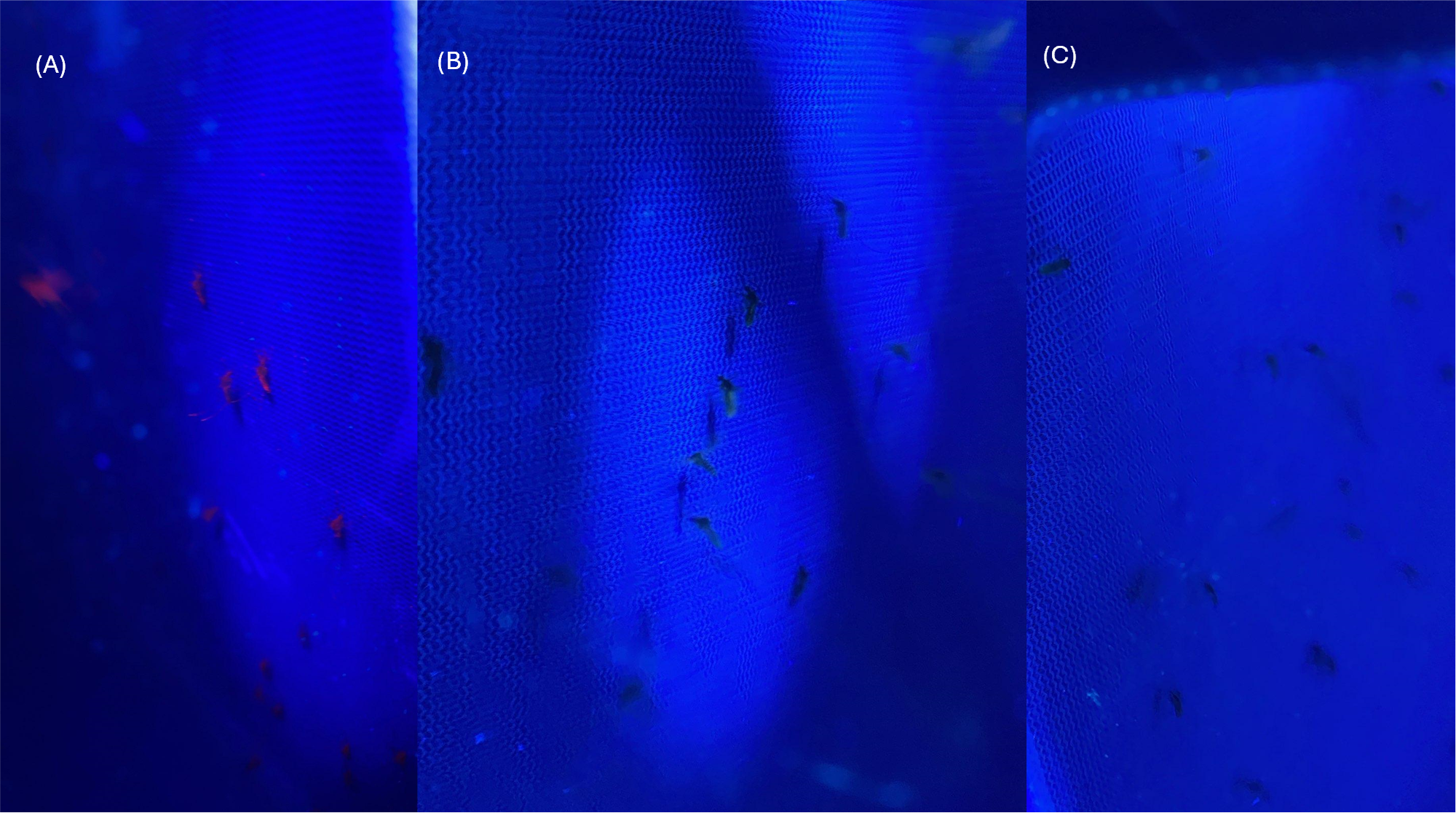


**Fig A. Photographs under ultraviolet light of mosquitoes sprayed (A) red, (b) green, or unsprayed (c).**


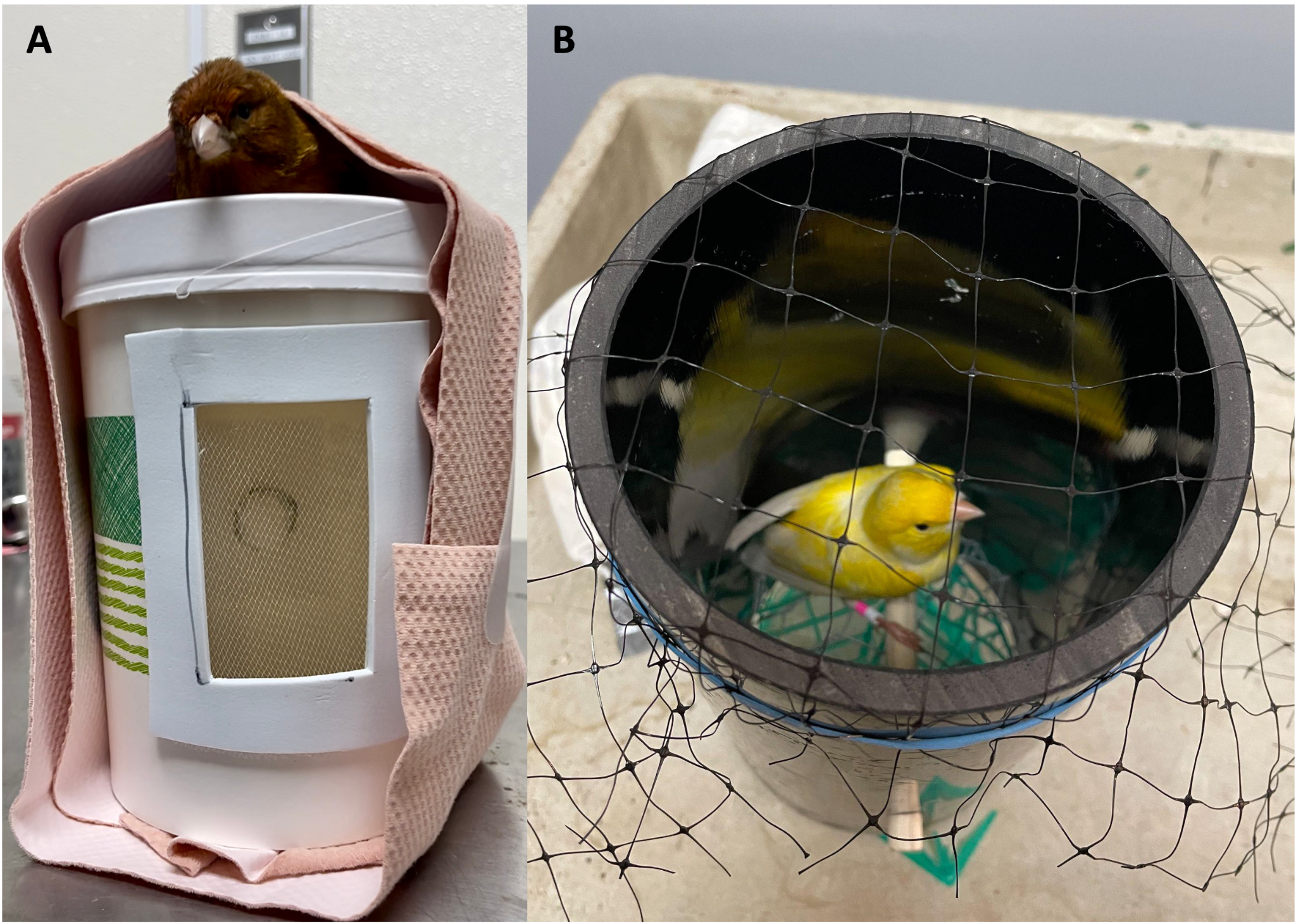


**Fig B. Mosquito feeding containers with A) restrained and B) unrestrained canaries.**  Feeding with both methods occurred in a room at 24 °C with the lights turned off. (A) The canary was restrained with a flexible athletic bandage on top of a 16-oz lidded paper container. The canary’s legs are pulled through holes in the lid so they are inside the container where mosquitoes can feed on them. Mosquitoes are placed into the container through an opening at the back using a mouth aspirator (Model 612, John W. Hock), which was then closed with a cotton ball to keep mosquitos inside. A mesh screen at the front of the container allowed monitoring of the feeding process. The container was placed inside a BugDorm mosquito cage (4S3030, W30 x D30 x H30cm). (B) The canary was unrestrained and sat on a wooden perch inside a vertical PVC cylinder (10 cm diameter x 30 cm height). The cylinder was elevated on a wire platform and capped with plastic netting. This allowed mosquitoes to access birds from above and below and prevented the bird from flying. The cylinder was then placed inside a mosquito cage (BugDorm-4S3030, W30 x D30 x H30cm) that was subsequently filled with mosquitoes.


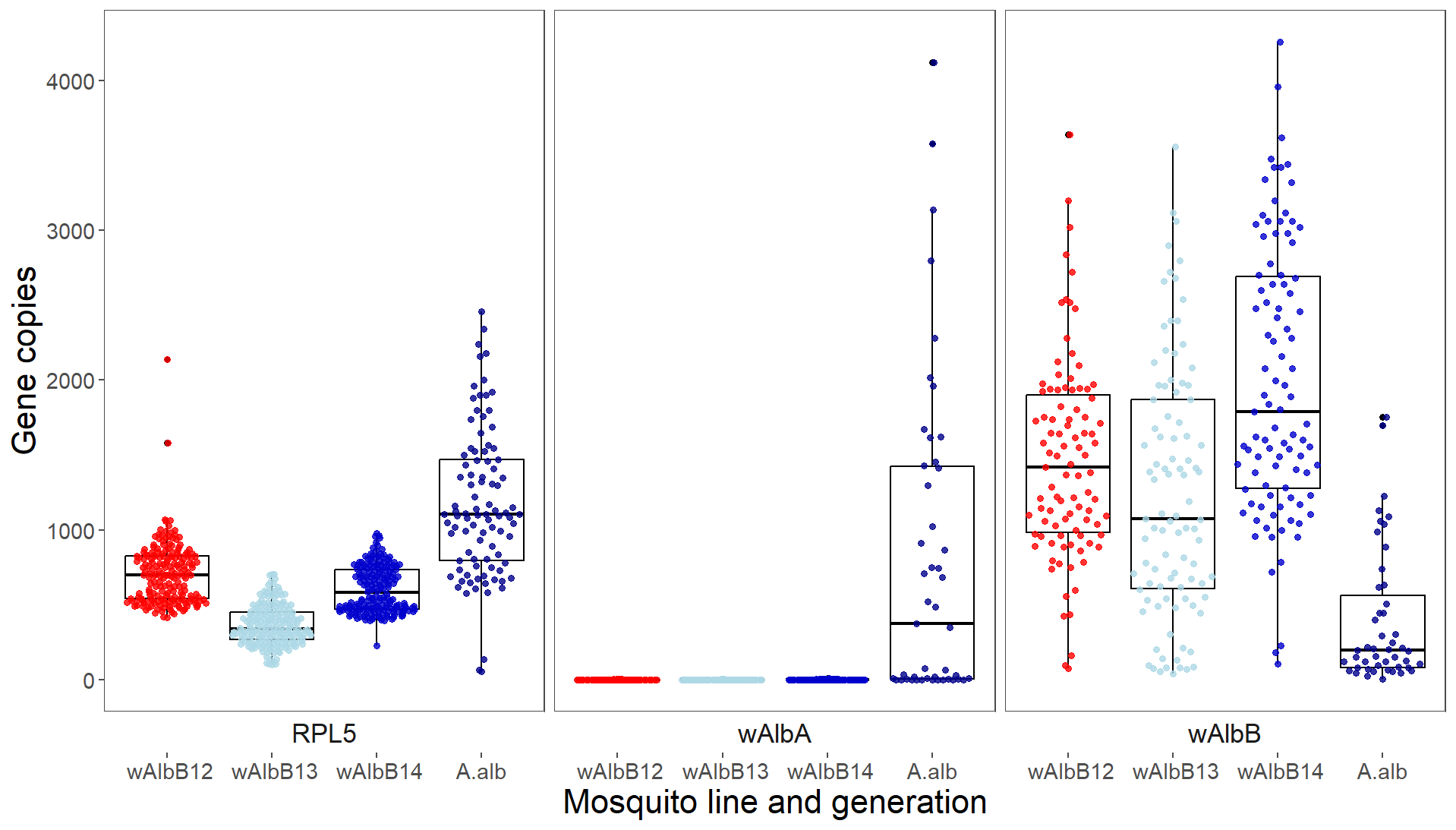


**Fig C. Number of gene copies per 20μL reaction for a mosquito housekeeping control gene RPL5, and Wolbachia gene WSP *w*AlbA and *w*AlbB in three generations of *Culex quinquefasciatus* transinfected with *Wolbachia* *w*AlbB (wAlbB12-wAlbB14, N = 95 males per generation), and an *Aedes albopictus* (*A. alb*) line (KLP) control (47 male and female mosquitoes).**


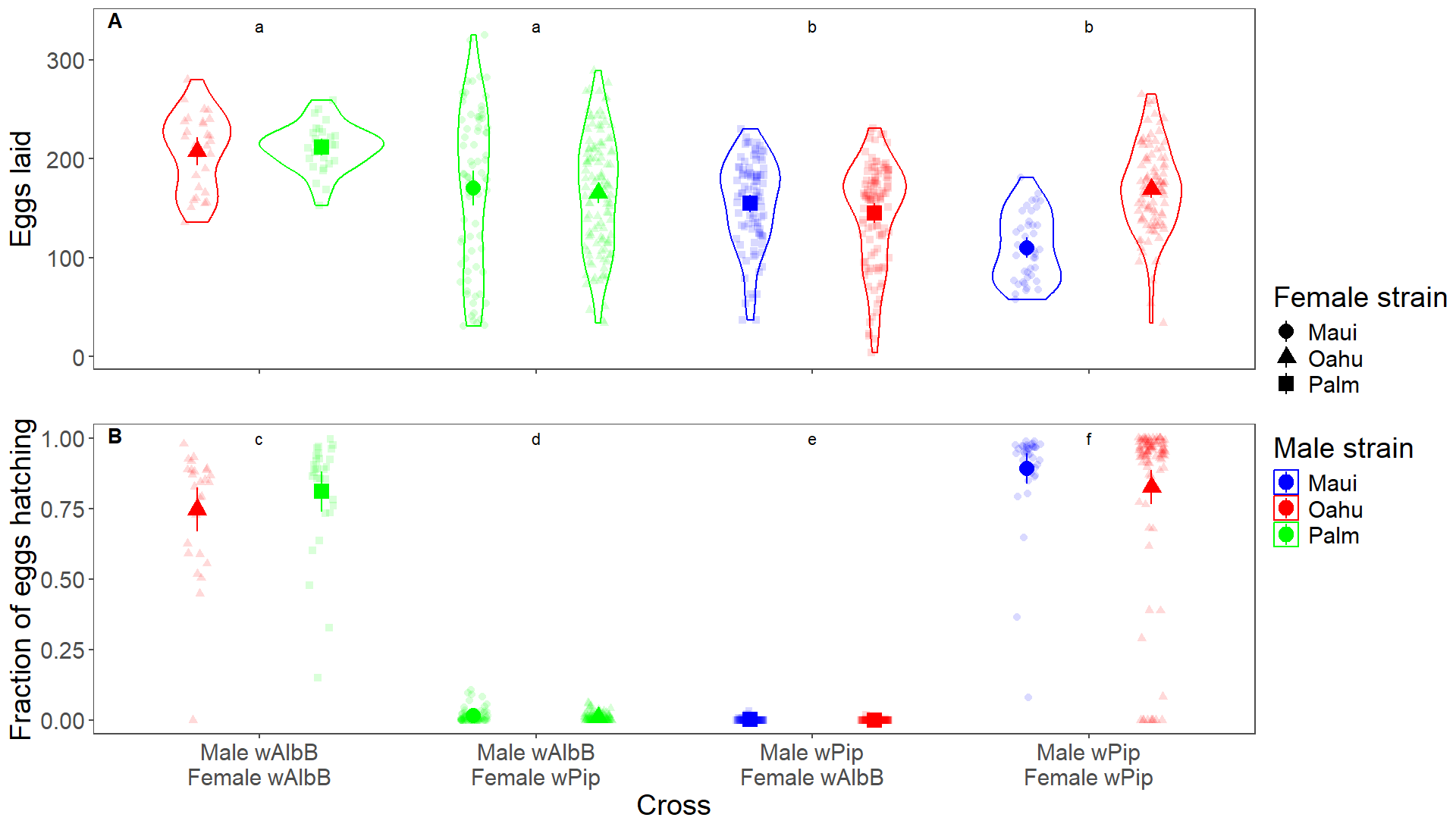


**Fig D. A) Fecundity and B) egg viability for individual *C. quinquefasciatus* mosquitoes with two *Wolbachia* types and from three genetic backgrounds in four different crosses.** Colored points show data for individual females. Black points and error bars show means and 95% CI. Different letters above the four crosses indicate which groups were significantly different in a model with male and female *Wolbachia* type and Female strain. The aggregated data in (B) is shown in Fig 1.


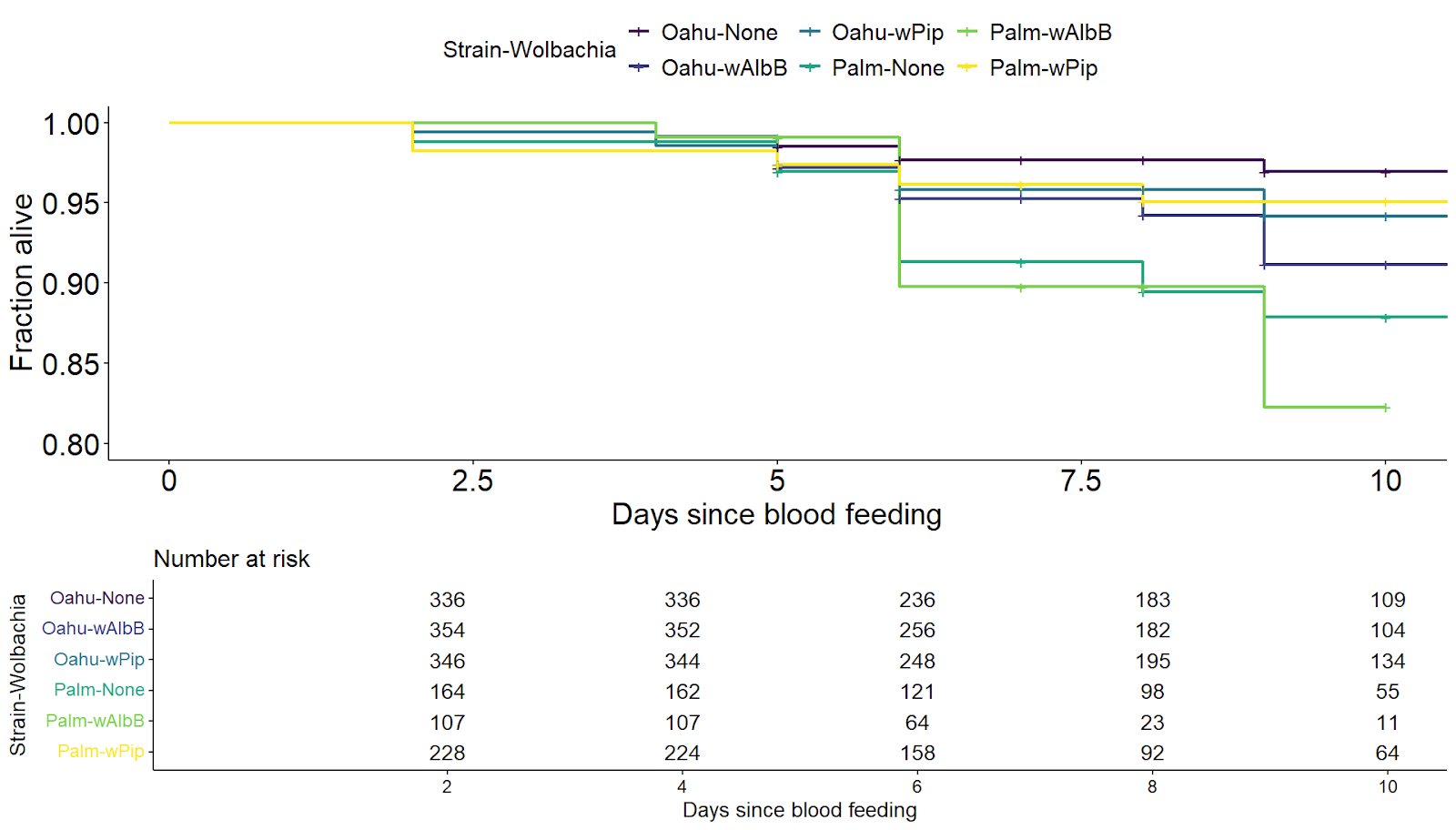


**Fig E. Survival of *C. quinquefasciatus* mosquitoes with two *Wolbachia* types or no *Wolbachia* (None) from two genetic backgrounds (Oahu, Palmyra).**


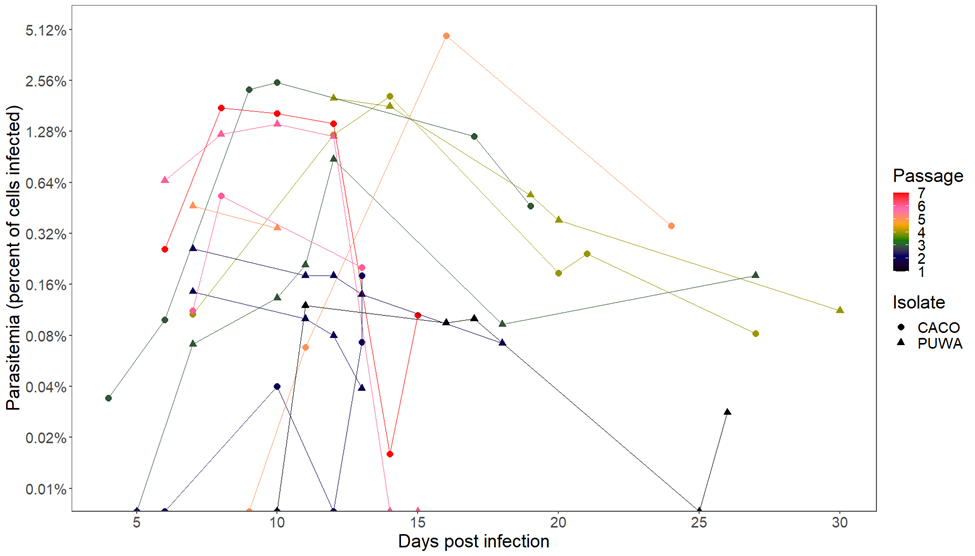


**Fig F. Parasitemias (percent of red blood cells infected) over time in eighteen domestic canaries infected with *Plasmodium relictum* GRW4.** Points show parasitemia estimates measured from thin blood smears using microscopy. Colors show the passage number for the malaria isolate and shape shows the isolate (CACO - Captain Cook; PUWA - Pu’u Wa'awa'a).


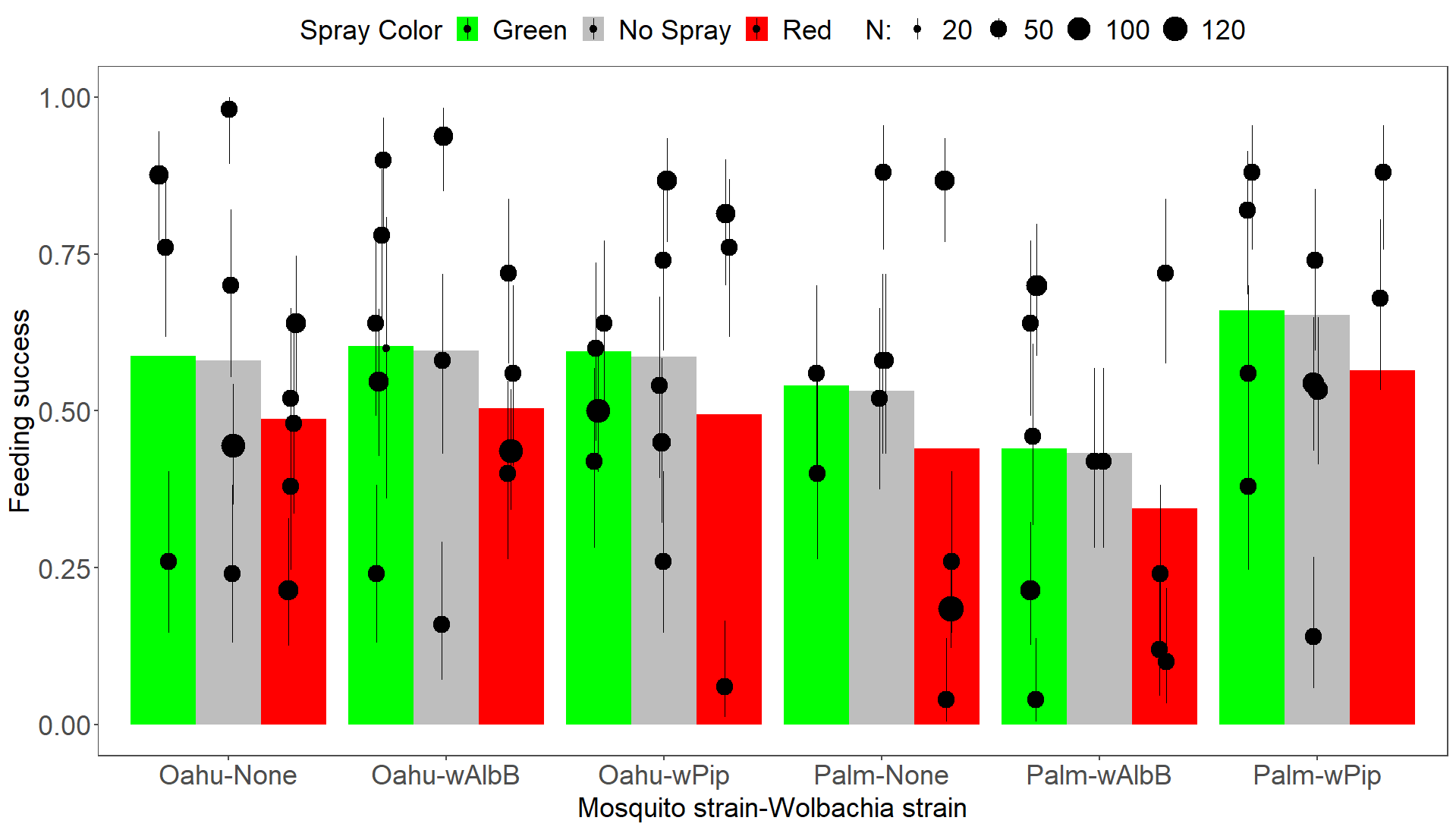


**Fig G. Feeding success for two strains of mosquitoes (Oahu and Palmyra (Palm)), each with two strains of *Wolbachia* (*w*AlbB, *w*Pip) or no *Wolbachia* (None), with two colors of spray (green or red) or no spray.** Points show values from individual experiments (with binomial 95% CIs) and the size of the points shows the sample size of mosquitoes (range 20-130). Bars show the fitted model for each of six strain-*Wolbachia* combinations, with the color indicating the spray color.


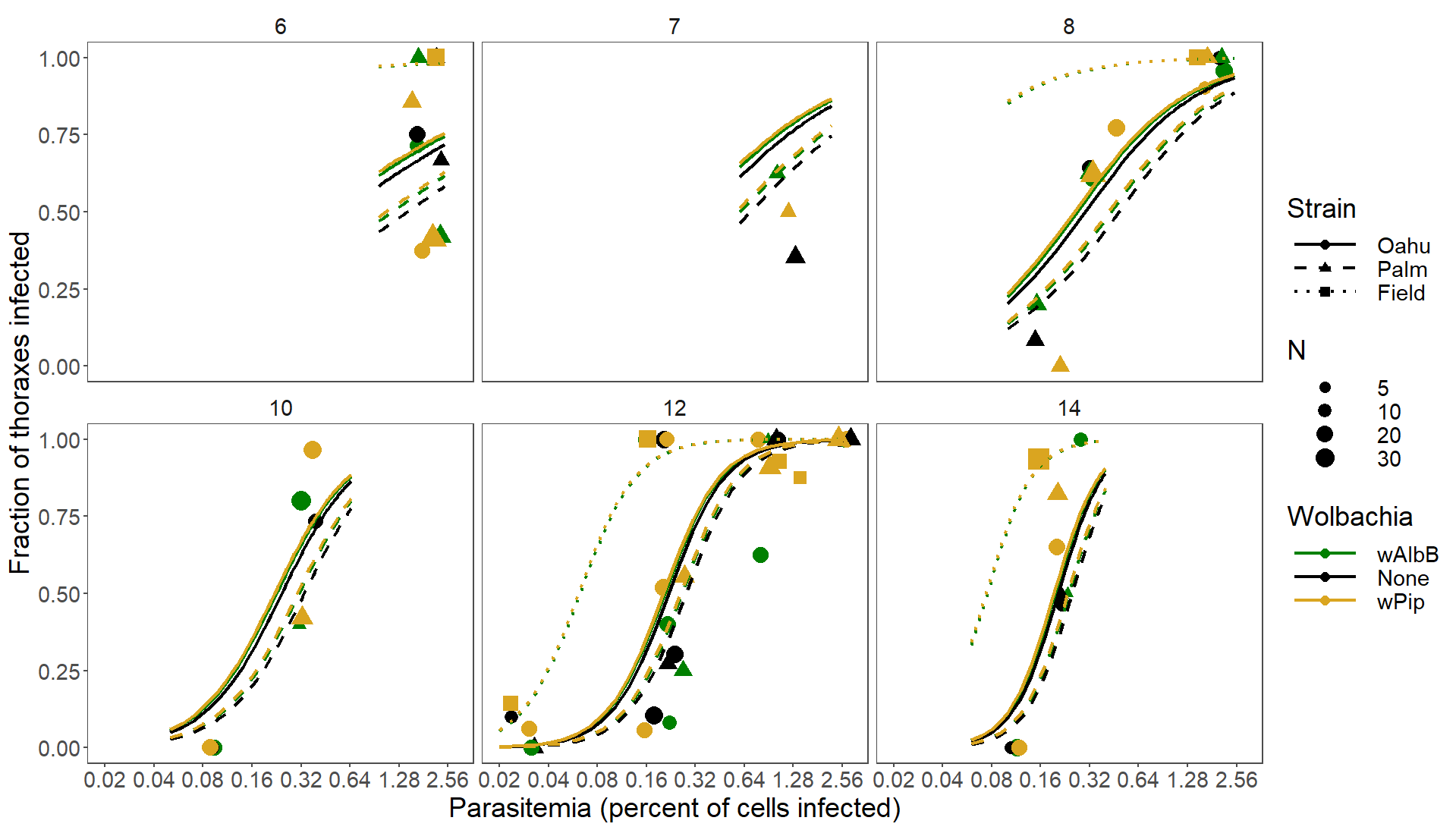


**Fig H. Fraction of thoraxes infected plotted against the parasitemia (percent of red blood cells infected) of the bird the mosquitoes fed upon (on a log_2_ scale).** The color shows the *Wolbachia* type in the mosquitoes (*w*AlbB, *w*Pip, or None), the symbol and line type show the mosquito strain (colonized Oahu or Palmyra mosquitoes, or Field-type mosquitoes from Hawaii island), the different panels show the days post-feeding when the mosquitoes were dissected (6-14 days), and the size of the points shows the sample size (range 4-39). The lines show the fitted model for each of the three *Wolbachia* types, which are often on top of each other and difficult to distinguish because there is no statistical support for differences among *Wolbachia* types. Points have been slightly jittered along the x-axis to aid in visualization. Fitted lines are only shown on panels where there was data for that day post-feeding for that mosquito strain (i.e., there were no Field-type mosquitoes tested on days 7 and 10 so no fitted lines are shown).


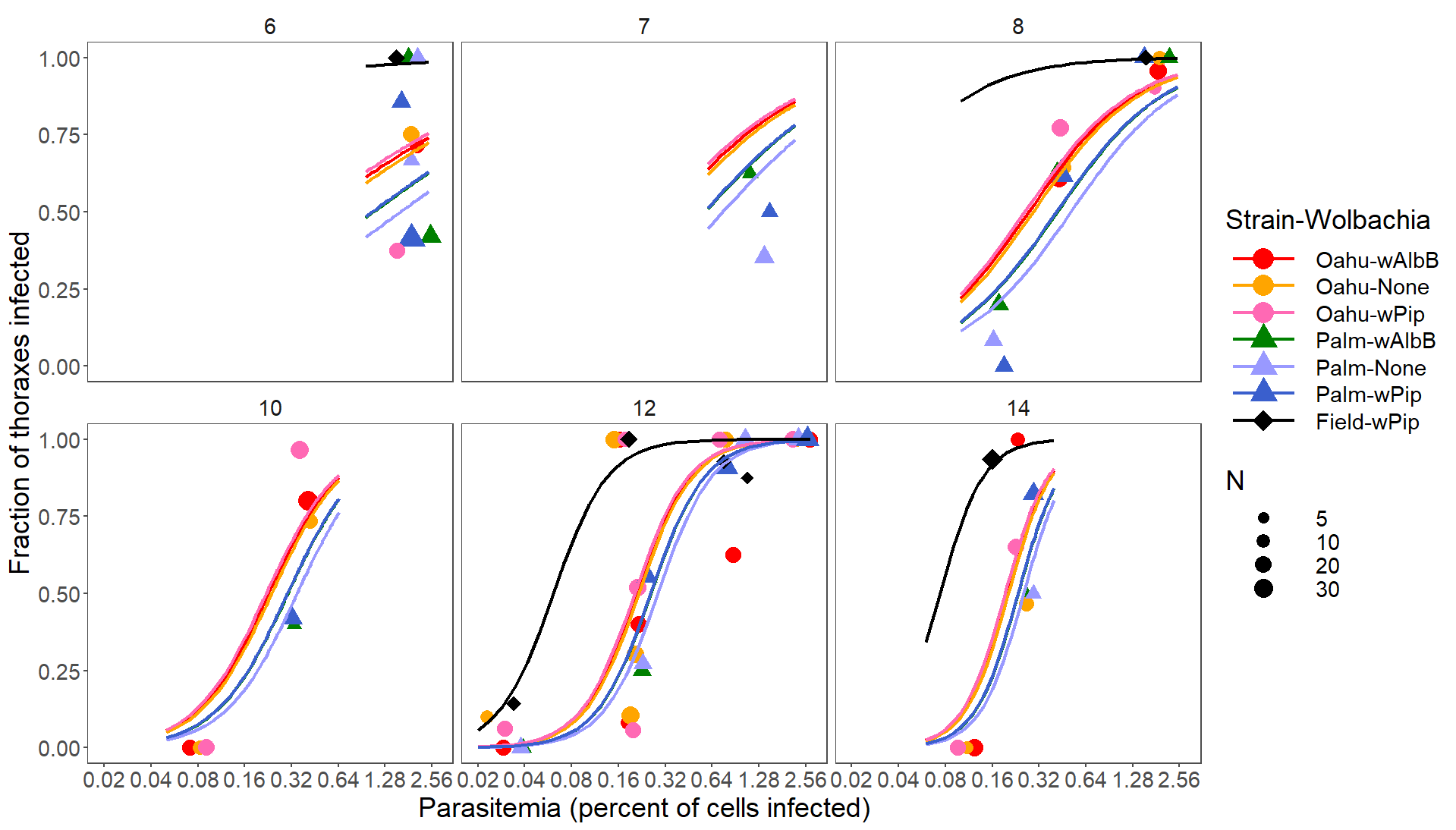


**Fig I. Fraction of thoraxes infected plotted against the parasitemia of the bird the mosquitoes fed on (on a log_2_ scale).** Color shows the mosquito strain (Oahu, Palm, Field) and *Wolbachia* strain (*w*Pip, *w*AlbB, none) pair, the different panels show the days post feeding when the mosquitoes were dissected (6-14), and the size of the points shows the sample size (range 4-39). The lines show the fitted model for each strain-*Wolbachia* pair; pairwise comparisons show differences among the three mosquito strains but no differences between *Wolbachia* strains within each mosquito strain. Points have been jittered along the x-axis to aid in visualization. Fitted lines are only shown on panels where there was data for that day post-feeding for that mosquito strain (i.e., there were no Field-type mosquitoes tested on days 7 and 10 so no fitted lines are shown).


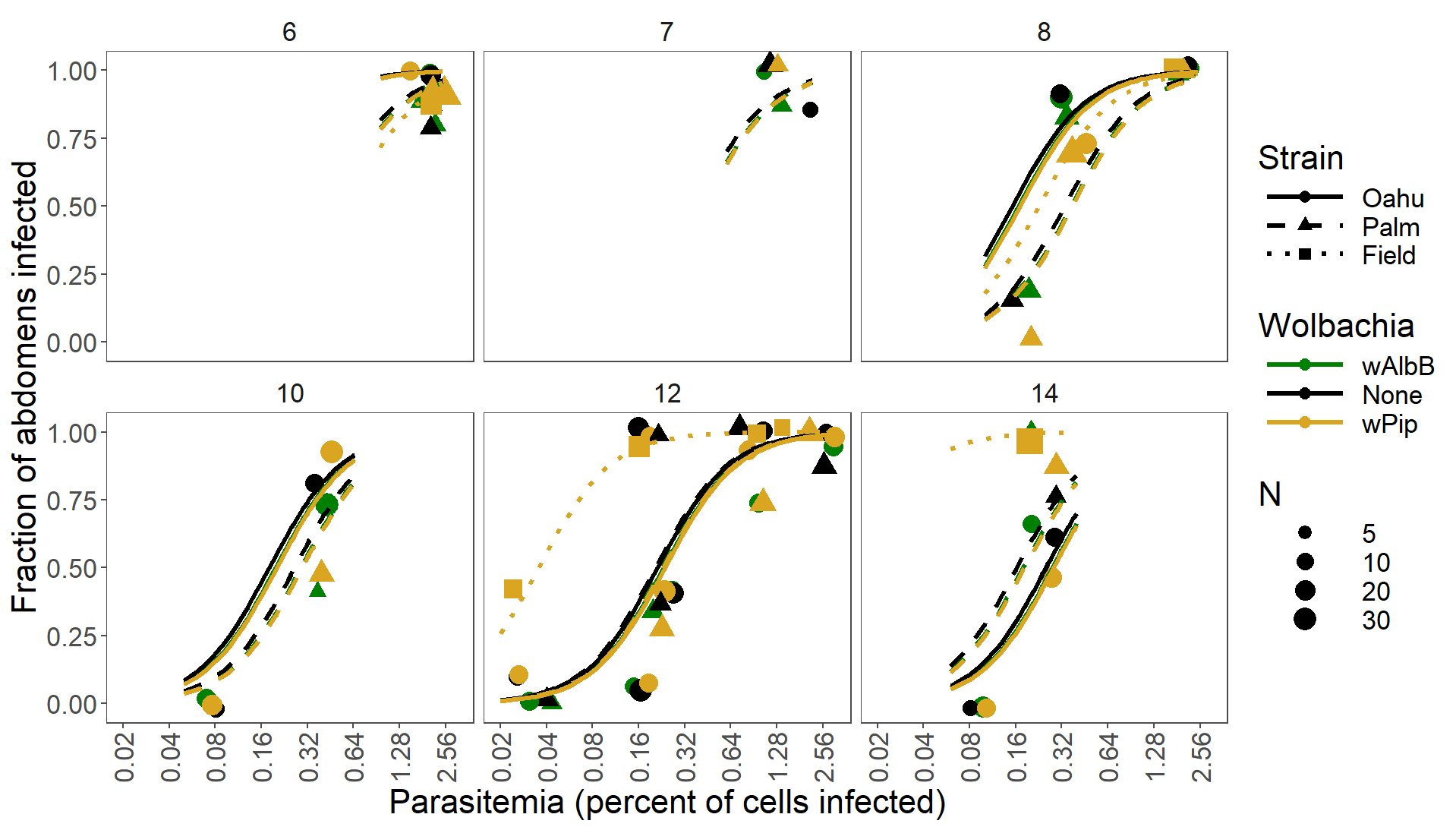


**Fig J. Fraction of abdomens infected plotted against the parasitemia (percent of red blood cells infected) of the bird the mosquitoes fed upon (on a log_2_ scale).** The color shows the *Wolbachia* type in the mosquitoes (*w*AlbB, *w*Pip, or None), the symbol and line type show the mosquito strain (colonized Oahu or Palmyra mosquitoes, or Field-type mosquitoes from Hawaii island), the different panels show the days post-feeding when the mosquitoes were dissected (6-14 days), and the size of the points shows the sample size (range 4-39). The lines show the fitted model for each of the three *Wolbachia* types, which are often on top of each other and difficult to distinguish because there is no statistical support for differences among *Wolbachia* types. Points have been slightly jittered along the x-axis to aid in visualization. Fitted lines are only shown on panels where there was data for that day post-feeding for that mosquito strain (i.e., there were no Field-type mosquitoes tested on days 7 and 10 so no fitted lines are shown).


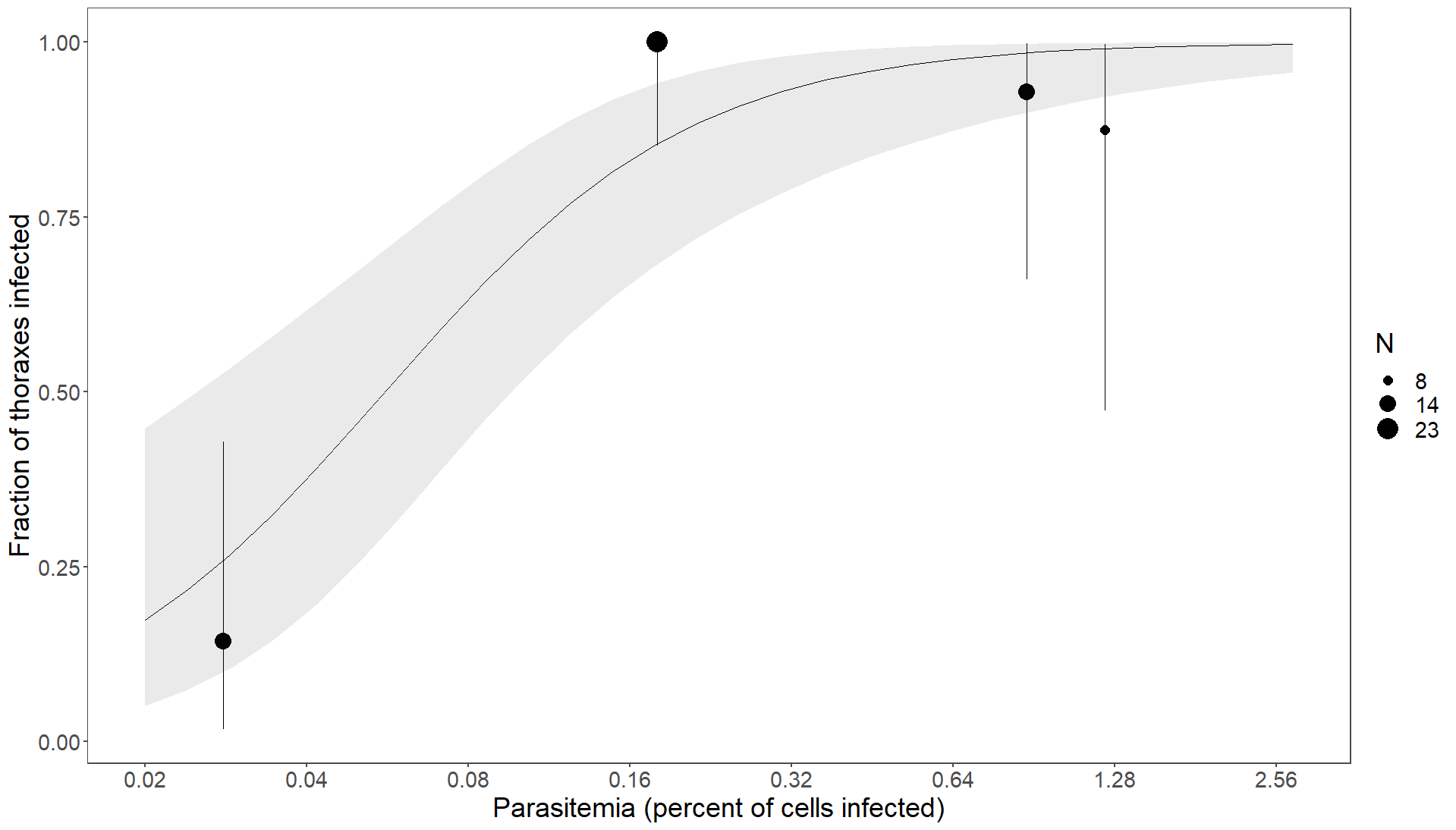


**Fig K. The fraction of thoraxes infected in Field *C. quinquefasciatus* twelve days after feeding, plotted against parasitemia (percent of red blood cells infected).** Points show the data and 95% CI binomial intervals, and the line and ribbon shows the fitted relationship (logit(Prevalence) = 4.37 + 1.52 * log(Percent parasitemia); slope SE = 0.39; Z-value = 3.89; P = 0.0001). This 95% CI of this slope (0.76, 2.28) is much lower and does not contain the slope of log parasitemia in a fit of the dataset without the Field strain (slope on day 12: -1.38 + 0.337 * 12 = 2.66). This slope (2.66) is similar to the estimated slope for the full dataset, which was primarily composed of data from the inbred mosquito strains (Table E: slope on day 12: -1.22 + 12 * 0.310 = 2.50).
